# Genomic‐wide analysis reveals *seven in absentia* genes regulating grain development in wheat (*Triticum aestivum* L.)

**DOI:** 10.1002/tpg2.20480

**Published:** 2024-06-05

**Authors:** Tao Chen, Yongping Miao, Fanli Jing, Weidong Gao, Yanyan Zhang, Long Zhang, Peipei Zhang, Lijian Guo, Delong Yang

**Affiliations:** ^1^ State Key Laboratory of Aridland Crop Science Gansu Agricultural University Lanzhou China; ^2^ College of Life Science and Technology Gansu Agricultural University Lanzhou China

## Abstract

Seven in absentia proteins, which contain a conserved SINA domain, are involved in regulating various aspects of wheat (*Triticum aestivum* L.) growth and development, especially in response to environmental stresses. However, it is unclear whether TaSINA family members are involved in regulating grain development until now. In this study, the expression pattern, genomic polymorphism, and relationship with grain‐related traits were analyzed for all TaSINA members. Most of the *TaSINA* genes identified showed higher expression levels in young wheat spikes or grains than other organs. The genomic polymorphism analysis revealed that at least 62 *TaSINA* genes had different haplotypes, where the haplotypes of five genes were significantly correlated with grain‐related traits. Kompetitive allele‐specific PCR markers were developed to confirm the single nucleotide polymorphisms in *TaSINA101* and *TaSINA109* among the five selected genes in a set of 292 wheat accessions. The *TaSINA101‐Hap II* and *TaSINA109‐Hap II* haplotypes had higher grain weight and width compared to *TaSINA101‐Hap I* and *TaSINA109‐Hap I* in at least three environments, respectively. The qRT‐PCR assays revealed that *TaSINA101* was highly expressed in the palea shell, seed coat, and embryo in young wheat grains. The TaSINA101 protein was unevenly distributed in the nucleus when transiently expressed in the protoplast of wheat. Three homozygous *TaSINA101* transgenic lines in rice (*Oryza sativa* L.) showed higher grain weight and size compared to the wild type. These findings provide valuable insight into the biological function and elite haplotype of *TaSINA* family genes in wheat grain development at a genomic‐wide level.

## Core Ideas

- At least 131 *TaSINA* genes were identified in the wheat genome.
- Most of the *TaSINA* genes showed high expression levels in young wheat spikes or grains.
- The haplotypes of five *TaSINA* genes were significantly correlated with grain‐related traits.
- Two kompetitive allele‐specific PCR markers were developed in *TaSINA101* and *TaSINA109* genes.
- The *TaSINA101*‐overexpression transgenic rice showed higher grain weight and size compared to the wild type.

**Abbreviations**
GL: grain length
GT: grain thickness
GW: grain width
KASP: kompetitive allele‐specific PCR
NLS: nuclear localization signal
SINA: seven in absentia
SINAT5: SEVEN IN ABSENTIA OF ARABIDOPSIS THALIANA5
TGW: 1000‐grain weight
UPS: ubiquitin‐proteasome system

## 1 INTRODUCTION

Wheat (*Triticum aestivum* L.) is one of the most important grain crops in the world, contributing about 20% of calories and proteins to the global human diet (Shiferaw et al., 2013). There is a major concern that the risk of a global food crisis is increasing due to the ever‐increasing population, extreme climate changes, and reduction in arable land (Curtis & Halford, 2014). It is also estimated that a 1.6% yield improvement per year will be required to meet the food needs of an increasing world population and harsh environments (Hatfield & Beres, 2019). Grain weight and size are the main determinants of wheat yield with higher heritability than other yield components, thus genetic improvement of grain traits is a viable approach to increase wheat yield (Li & Li, 2016). One way to breed ideal wheat varieties is to discover genes related to grain weight and size and deploy these genes through molecular breeding (Thomelin et al., 2021).

The E3 ubiquitin ligase is essential for recognizing target proteins and promoting substrate degradation via the ubiquitin‐proteasome system (UPS) (Al‐Saharin et al., 2022; Shu & Yang, 2017). Therefore, E3 ubiquitin ligases have been extensively studied, especially for large families such as RING‐type E3 ubiquitin ligases (Qin et al., 2014; Simmonds et al., 2016; Song et al., 2007; Wang & Deng, 2011; Zhang et al., 2018). In our previous study, we found UPS is one of the major KEGG enrichment pathways in the regulation of wheat grain via meta‐quantitative trait locus analysis with 394 reported QTLs for 1000‐grain weight (TGW), where several *seven in absentia* (*SINA*) family members were mined as the major candidate genes for TGW (Miao et al., 2022). SINA proteins possess a conserved SINA domain and always with a functional RING domain, and thus are generally considered as a subfamily of RING‐type E3 ubiquitin ligases. The RING domain in SINA proteins binds to the E2 ubiquitin‐binding enzyme, and the SINA domain serves to the formation of homodimer or heterodimer and recognition of the target protein for subsequent degradation by the 26S proteasome (Den Herder et al., 2012; Hu & Fearon, 1999; J. Ma et al., 2023; M. Wang et al., 2008).

As the first member of SINA proteins that were functionally characterized in plants, SEVEN IN ABSENTIA OF ARABIDOPSIS THALIANA5 (SINAT5) serves as a versatile regulator to participate in the regulation of lateral root development and flowering, and negatively regulated BR signaling (Park et al., 2010, 2007; Xie et al., 2002; Yang et al., 2017). SINAT2, another SINA member, was found to mediate the carotenogenesis of leaves via interacting with AtRAP2.2 in *Arabidopsis thaliana* (Xie et al., 2002). In tomato (*Solanum lycopersicum* L.), SlSINA2 and SlSINA5 co‐regulated the chlorophyll levels in the leaf and changed the flower structure (Wang et al., 2018). SINA proteins also play important roles in response to abiotic and biotic stress in plants. SINA2 positively regulated drought tolerance in an ABA‐dependent manner in Arabidopsis (Bao et al., 2014), while OsDIS1 was identified as a negative regulator in the drought stress tolerance in rice (*Oryza sativa* L.) (Ning et al., 2011). In tomato, SINA proteins participate in the regulation of immunity response via a synergistic or antagonistic relationship. When overexpressed in *Nicotiana benthamiana* leaves, SlSINA4 triggered cell death, whereas SlSINA1/2/3/5/6 suppressed it (Wang et al., 2018). Besides, only SlSINA3, but not SlSINA1/2/4/5/6, interacted with SlNAC1 to promote its degradation through the ubiquitination pathway, thereby positively regulating the defense response (M. Miao et al., 2016). The SINA proteins only containing a conserved SINA domain and lacking a RING domain serve their functions as a competitor to other SINA proteins possessing both two domains. For example, SINA proteins control ATG1 and ATG13 stability and autophagy dynamics by modulating ATG13 ubiquitylation in Arabidopsis (Hua et al., 2020). Among them, SINAT1 and SINAT2 interacted with ATG13 to ubiquitin it and subsequently affected its stability, whereas SINAT5‐S1 and SINAT6 both lacking the RING domain competitively interacted with ATG13 to induce autophagosome biogenesis under starvation conditions (Hua et al., 2020). Therefore, SINA family proteins perform diverse roles in plant growth and response to abiotic and biotic stress via their SINA domain with or without the RING domain.

In wheat, a total of 15 TaSINA proteins have been selected in the wheat genome (IWGSC RefSeq v2.1) through the conserved RING domain at the N‐terminus and SINA domain at the C‐terminus to define as SINA protein (J. Ma et al., 2023). Recently, a total of 141 TaSINA family members were identified in bread wheat using SINA sequences characterized in Arabidopsis and tomato (Roche et al., 2023). The most identified and cloned *TaSINA* genes were found to participate in abiotic stress tolerance in wheat, especially in drought stress so far. For example, TaDIS1, a homolog of OsDIS1, affected grain germination and early seedling growth and served as a negative regulatory in response to drought stress (Liu et al., 2018). A recent study showed that TaDIS1 interacted with TaSTP to degrade it via the ubiquitin pathway (Lv et al., 2020). TaSINA2B, another TaSINA protein, has been found to positively regulate drought tolerance by promoting root growth via interacting with TaSINA1D (J. Ma et al., 2023). A *TaSINA* gene cloned at QTL *qYDH.3BL* on chromosome 3B in bread wheat was associated with increased yield in hot and dry climates, which elite alleles have been positively selected in breeding programs in Mexico and Australia (Thomelin et al., 2021). TaSIRFP‐3A and TaSIRFP‐3B were found to participate in the regulation of sucrose metabolism and cold tolerance in wheat (Park & Jang, 2020). Overexpression of *TaSIRFP‐3A* and *TaSIRFP‐3B* in Arabidopsis displayed hypersensitivity phenotypes under cold stress with an accumulation of reactive oxygen species (Park & Jang, 2020). Meanwhile, the transgenic plants also showed delayed plant growth by affecting sucrose synthase (Park & Jang, 2020). Collectively, the studies of *TaSINA* family genes were mainly focused on abiotic stress, and the knowledge on the roles of *TaSINA* family genes in wheat development is still limited, compared with vast studies of *SINA* genes in other plants.

In this study, to ascertain the roles of *TaSINA* family members in the regulation of wheat growth, especially in grain development, the expression pattern of *TaSINA* family members was investigated through the wheat RNA‐Seq database and confirmed with qRT‐PCR results. The elite haplotypes of *TaSINA* genes in grain development were identified using the data from the WheatUnion platform and published studies. Then, the kompetitive allele‐specific PCR (KASP) markers in the genomic sequences of *TaSINA101* and *TaSINA109* were developed, and association analysis was performed between different haplotypes with grain weight and size in multiple environments. The phenotype of *TaSINA101* transgenic lines in rice was also observed to confirm its function in grain development. This study aims to provide a good insight into exploring the biological roles and elite haplotype of *TaSINA* family genes in the development of wheat grain at the genomic‐wide level.

**Core Ideas**

- At least 131 *TaSINA* genes were identified in the wheat genome.
- Most of the *TaSINA* genes showed high expression levels in young wheat spikes or grains.
- The haplotypes of five *TaSINA* genes were significantly correlated with grain‐related traits.
- Two kompetitive allele‐specific PCR markers were developed in *TaSINA101* and *TaSINA109* genes.
- The *TaSINA101*‐overexpression transgenic rice showed higher grain weight and size compared to the wild type.

## 2 MATERIALS AND METHODS

### 2.1 Plant materials, growth conditions, and grain traits measurement

The common wheat cultivar Jinmai 47 (JM47) was used for analyzing the expression level of *TaSINA* genes via qRT‐PCR and cloning the *TaSINA101* gene. Three wheat populations were used as follows. Population I was a wheat population of 681 wheat accessions, which genomic information acquired from the WheatUnion (http://wheat.cau.edu.cn/WheatUnion/?language=en) platform (Brinton & Uauy, 2019; Cheng et al., 2019; Hao et al., 2020; J. Wang et al., 2020; Y. Zhou et al., 2020) (Table S1) and used for detecting the genomic polymorphisms of *TaSINA* family genes. Population II comprised 122 wheat accessions whose grain traits‐related data have been published from a previous study (L. Ma et al., 2016) (Table S2). The genomic information of these accessions is also available on the WheatUnion platform, and hence they were also included in Population I. Population II was used for association analysis to discover the relationship between *TaSINA* family genes and grain‐related traits. Population III was a wheat natural population of 292 accessions used for detecting nucleotide polymorphisms of *TaSINA101* and *TaSINA109* with KASP functional markers and performing the association analysis of different haplotypes with grain traits (Table S3). Zhonghua 11 (ZH11) was used as a recipient rice line for genetic transformation.

JM47 and ZH11 seeds were grown at 26°C under long‐day conditions (16‐h light/8‐h dark) in the greenhouse. Population III was planted in five environments at Tongwei farm station (105°19′E, 35°11′N, 1750 m ASL) in Gansu, China, during the 2020–2021 and 2021–2022 growing seasons, Zhuanglang farm station (105°58′E, 35°21′N, 2110 m ASL) in Gansu, China, during 2021–2022 and 2022–2023, and Zhongliang farm station (105°53′E, 34°34′N, 1550 m ASL) in Gansu, China, during 2021–2022 and 2022–2023. Each line was planted in a 1‐m, six‐row plot with a row spacing of 20 cm and 30 seeds per row. Fifteen plants in the middle of each plot were randomly sampled for phenotyping at the maturity stage. After air drying, 200 seeds of each line were randomly selected to measure the grain length (GL), grain width (GW), and TGW using a SC‐G image analysis system (Hangzhou Wanshen Detection Technology Co., Ltd.). The grain thickness (GT) was measured with vernier caliper.

### 2.2 Identification of wheat TaSINA members

The wheat genomic data, protein sequences, and annotation files were obtained from the *Ensembl* Plants database (http://plants.ensembl.org). The SINA conserved structural domain HMMER file (PF03145) was downloaded from the InterPro database (https://www.ebi.ac.uk/interpro/) (Paysan‐Lafosse et al., 2023) and used as a query sequence to search the wheat protein sequences using the HMMER 3.0 software (https://www.ebi.ac.uk/Tools/hmmer/search/hmmsearch) (Finn et al., 2006) program to obtain the wheat TaSINA candidate proteins (threshold *E *< 1e‐5). These candidate proteins were then verified by NCBI‐CDD (https://www.ncbi.nlm.nih.gov/Structure/bwrpsb/bwrpsb.cgi), InterPro, and SMART (http://smart.embl‐heidelberg.de/) platform (Finn et al., 2006; Letunic et al., 2021; Paysan‐Lafosse et al., 2023; Yang et al., 2020). The phylogenetic tree of TaSINA members was constructed using MEGA11 software (https://www.megasoftware.net/) (Tamura et al., 2021), and the neighbor‐joining algorithm was used to build the tree with 1000 times of bootstrap replicates. The conserved motifs and domains of TaSINA proteins were identified using the MEME (http://meme.nbcr.net/meme) and SMART platform, respectively. An accurate exhibition was manually performed based on the presence of the conserved motifs consisting in SINA and RING domains and visualized using TBtools software (Chen et al., 2020).

### 2.3 The expression pattern analysis of *TaSINA* family genes

RNA‐Seq data of Chinese Spring was downloaded through the expVIP (http://www.wheat‐expression.com/) platform (Borrill et al., 2016) at different organs and growth stages to analyze the expression pattern of *TaSINA* family genes. The expression level of *TaSINA* genes was assessed by TPM values (Wagner et al., 2013) and normalized based on TBtools software (Chen et al., 2020) for visualized mapping.

For qRT‐PCR analysis to confirm the results from RNA‐Seq data of Chinese Spring, the root, stem, leaf, and spike during booting, spike at the anthesis stage, and grain at 5, 10, 15, and 20 days after anthesis were collected from wheat cultivar JM 47. The total RNA was extracted from these organs and then synthesized to cDNA with reverse transcriptase. Three biological replicates were set for each sample using the TIANGEN FastReal qPCR PreMix (SYBR Green) for gene expression experiments. The qRT‐PCR reaction was performed in a LongGene Q2000A instrument (Hangzhou LongGene Scientific Instruments Co., Ltd.) with the following amplification procedures: 95°C for 10 s, 58°C for 10 s, 72°C for 20 s, total 40 cycles. Gene expression level in the root was used as a control, the *TaACTIN* gene was used as a reference, and the relative gene expression level was determined by the 2^−ΔΔCT^ method (Schmittgen & Livak, 2008). The primer sequences are shown in Table S4.

### 2.4 KASP marker development

The SNP (T/G) at position 190 bp downstream away from the initiation codon of *TaSINA101* and the SNP (T/C) at position 810 bp away from that of *TaSINA109* were converted into KASP markers for genotyping wheat accessions in the population III. The KASP markers were designed on the WheatOmics 1.0 platform (http://wheatomics.sdau.edu.cn/) (S. Ma et al., 2021). The primers were designed carrying the junction sequence of the fluorescent moiety FAM (6‐carboxy‐fluorescein) and HEX (Hexachloro fluorescein) at the 5′ terminals (Table S4). The KASP assays were tested in a 96‐well plate, with 4 µL reaction volume containing 2 µL of 2 × KASP master mix, 1 µL of SNP primer mix (4×), and 1 µL of DNA template. Touchdown PCR cycling was performed as follows: 94°C for 15 min, followed by 10 touchdown cycles (94°C for 20 s, touchdown 61°C, drop 0.6°C per cycle for 40 s), and then 35 cycles of amplification at 94°C for 20 s, and 55°C for 45 s. The fluorescence signal was detected and read using Kluster Caller software in the FLUOstar Omega instrument (LGC Genomics Ltd.).

### 2.5 The genomic polymorphism analysis and association analysis of *TaSINA* family genes

The WheatUnion database was used to obtain the variant loci of *TaSINA* family genes in population I based on the haplotype network module to view the genotypes (Brinton & Uauy, 2019; Cheng et al., 2019; Hao et al., 2020; J. Wang et al., 2020; Y. Zhou et al., 2020). The upstream and downstream extension lengths were set to 2000 bp. The data were downloaded via the variant information query module.

The TGW data of wheat accessions in population II and grain‐related phenotypic data of wheat accessions in population III were both used for association analysis with the TASSEL5.1 software (Tables S2 and S3).

### 2.6 The grain‐specific expression distribution and subcellular localization of TaSINA101

The ovary at the anthesis stage, the seed coat at 5 days after anthesis, the seed coat, endosperm, embryo, and palea shell at 15 days after anthesis were collected from wheat variety JM47 to determine the specific expression location of *TaSINA101* gene in wheat grain. The detailed qRT‐PCR program was described above.

The CDS region of *TaSINA101* (TraesCS3B02G052800) was amplified by specific primers (Table S4), and inserted into pEarleyGate103 by using homologous recombinase to generate the construct pro35S::TaSINA101‐GFP. The protoplasts of wheat were isolated and transfected as described previously (Luo et al., 2022) and then observed by microscope. According to a previous report (F. Zhao et al., 2017), the nuclear localization signal (NLS) was fused with far‐red fluorescent reporter protein mKATE to confirm the nucleus location of TaSINA101.

### 2.7 Development of *TaSINA101*‐overexpression transgenic rice

The construct pro35S::TaSINA101‐GFP was introduced into *Agrobacterium tumefaciens* strain EHA105. Rice transformation was conducted by the *A. tumefaciens*‐mediated method (Biorun Biosciences Co., Ltd.). The positive transgenic plants were identified by Basta, and the homozygote transgenic lines were screened by PCR with *TaSINA101*‐specific primers used for CDS amplification and finally obtained by continuous self‐crossing of single‐locus transgenic plants.

### 2.8 Statistical data analysis

One‐way analysis of variance was performed to analyze the significance differences using the statistical software of SPSS version 22.0 (IBM Corporation).

## 3 RESULTS

### 3.1 Identification and classification of *TaSINA* family genes in wheat

**FIGURE 1:**
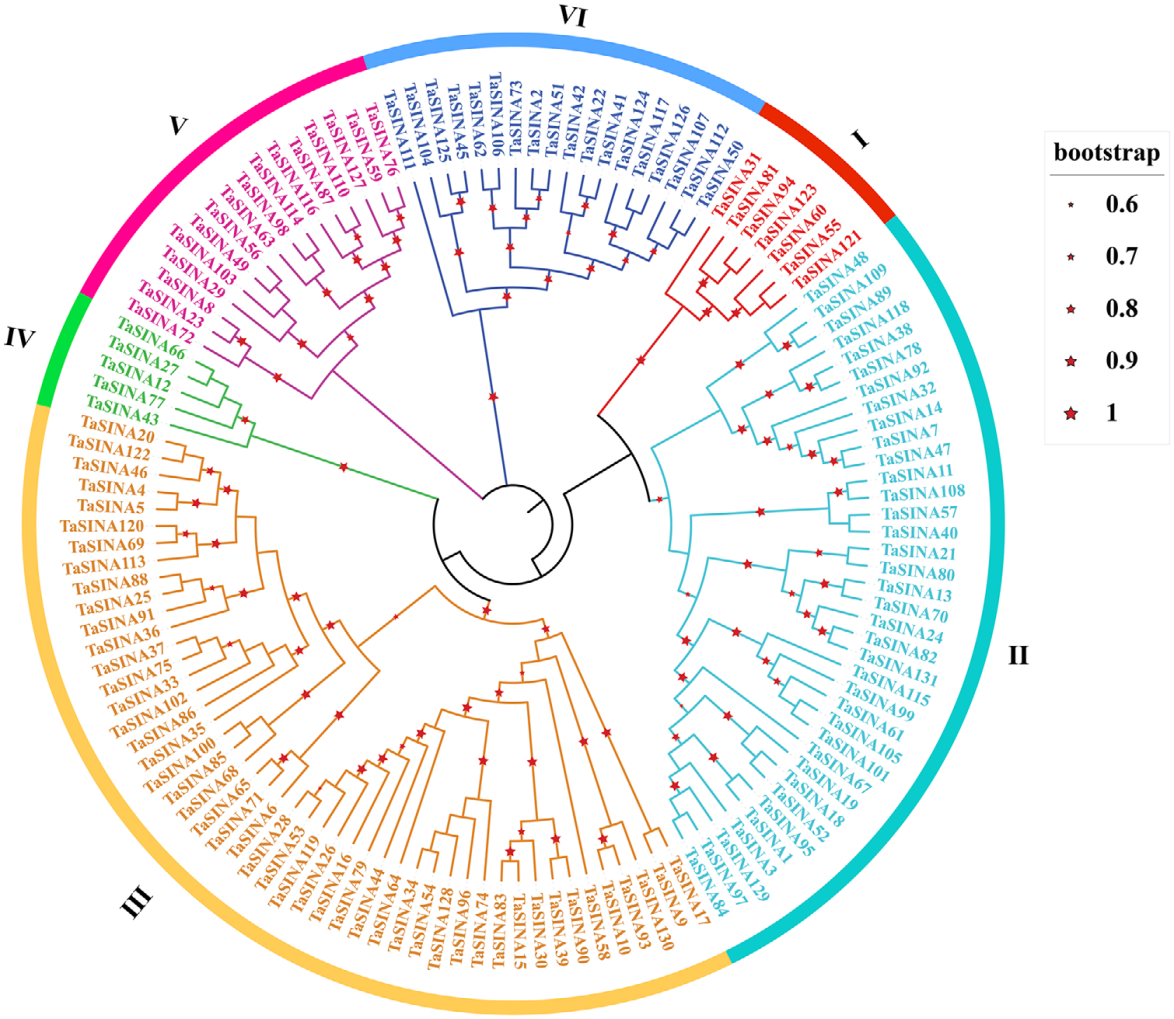
Phylogenetic tree analysis of TaSINA proteins. Different color blocks indicate different subclasses of the *TaSINA* family members.

**FIGURE 2:**
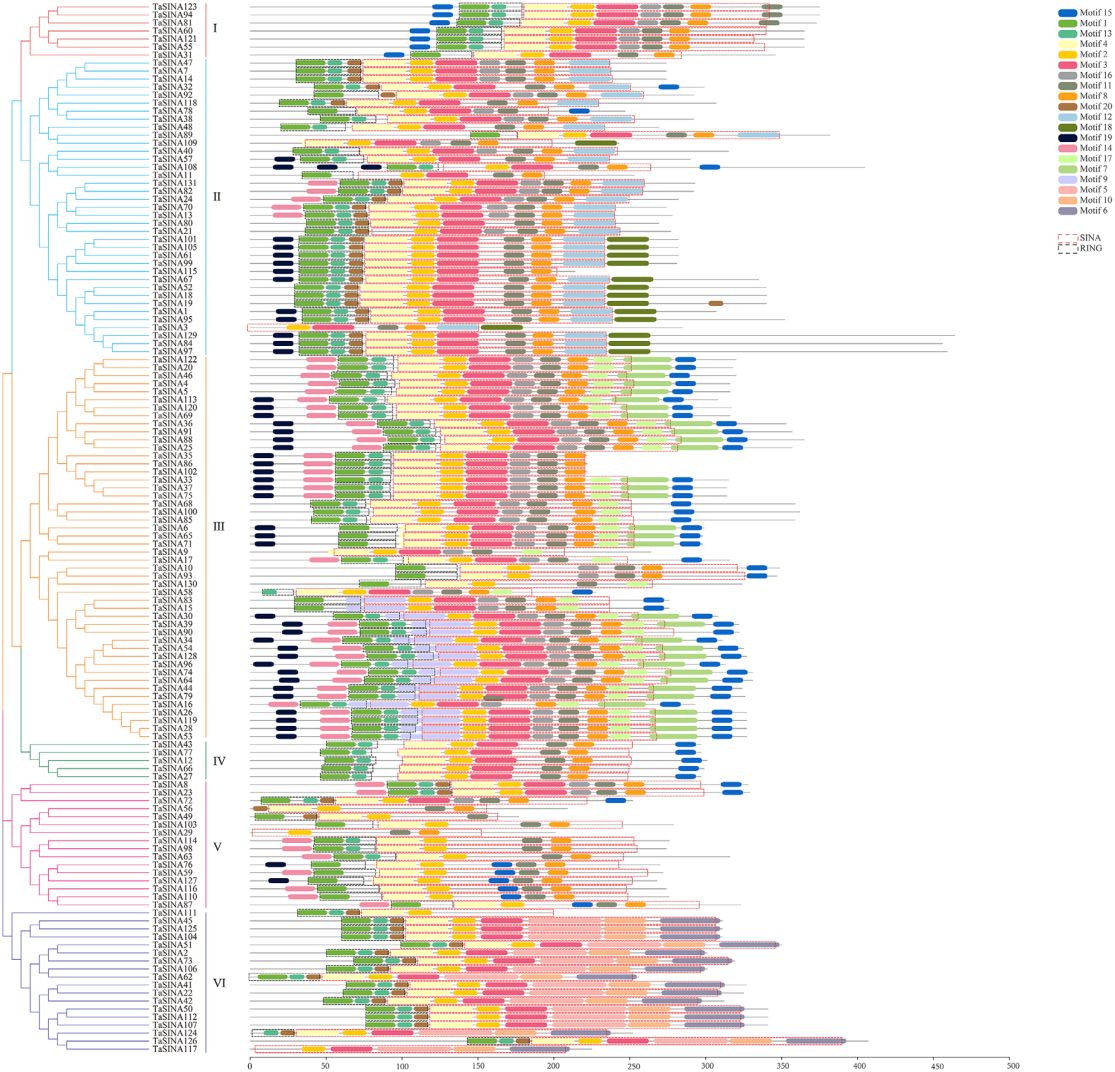
Phylogenetic relationships, conserved motifs, and functional domain of the TaSINA proteins. The phylogenetic tree for each subgroup of the TaSINA family is indicated with different colors. The 20 conserved motifs under investigation are represented in a range of colors. The RING and SINA domains are distinguished by red and black dotted lines, respectively.

A total of 131 candidate genes were identified, which transcript expression levels can be detected in previous RNA‐seq data (Table S5), according to the conserved SINA domain existed in their encoding proteins. These proteins were named TaSINA1‐131 in this study for better understanding (Table S6). The length of coding regions of 131 *TaSINA* family members ranged from 534 to 1395 bp, and the putative molecular weight of their encoded proteins ranged from 19.78 to 52.04 kD (Table S6). To evaluate the evolutionary relationships of these TaSINA proteins, the full‐length amino acid sequences of 131 TaSINA proteins were used for multiple sequence alignment and construction of a Neighbor‐Joining phylogenetic tree. The results showed that 131 TaSINA family members could be divided into six subclades (Figure 1). Intriguingly, 16 ancestral TaSINA proteins (TaSINA2, 22, 41, 42, 50, 51, 62, 73, 104, 106, 107, 112, 117, 124–126) identified in a previous report were all clustered in subclade VI. The conserved motif and domain of TaSINA proteins were further analyzed to investigate the structural diversity of TaSINA proteins in different subclades (Figure 2). Among them, all TaSINA proteins contained a SINA domain, whereas several members did not contain any conserved motif possessed in the RING domain, including TaSINA3 and TaSINA109 in subclade II, TaSINA9 in subclade III, TaSINA29 in subclade V, and TaSINA117 in subclade VI. This indicated that these proteins could perform their biological functions only with the SINA domain.

### 3.2 Expression patterns of *TaSINA* family genes in wheat

**FIGURE 3:**
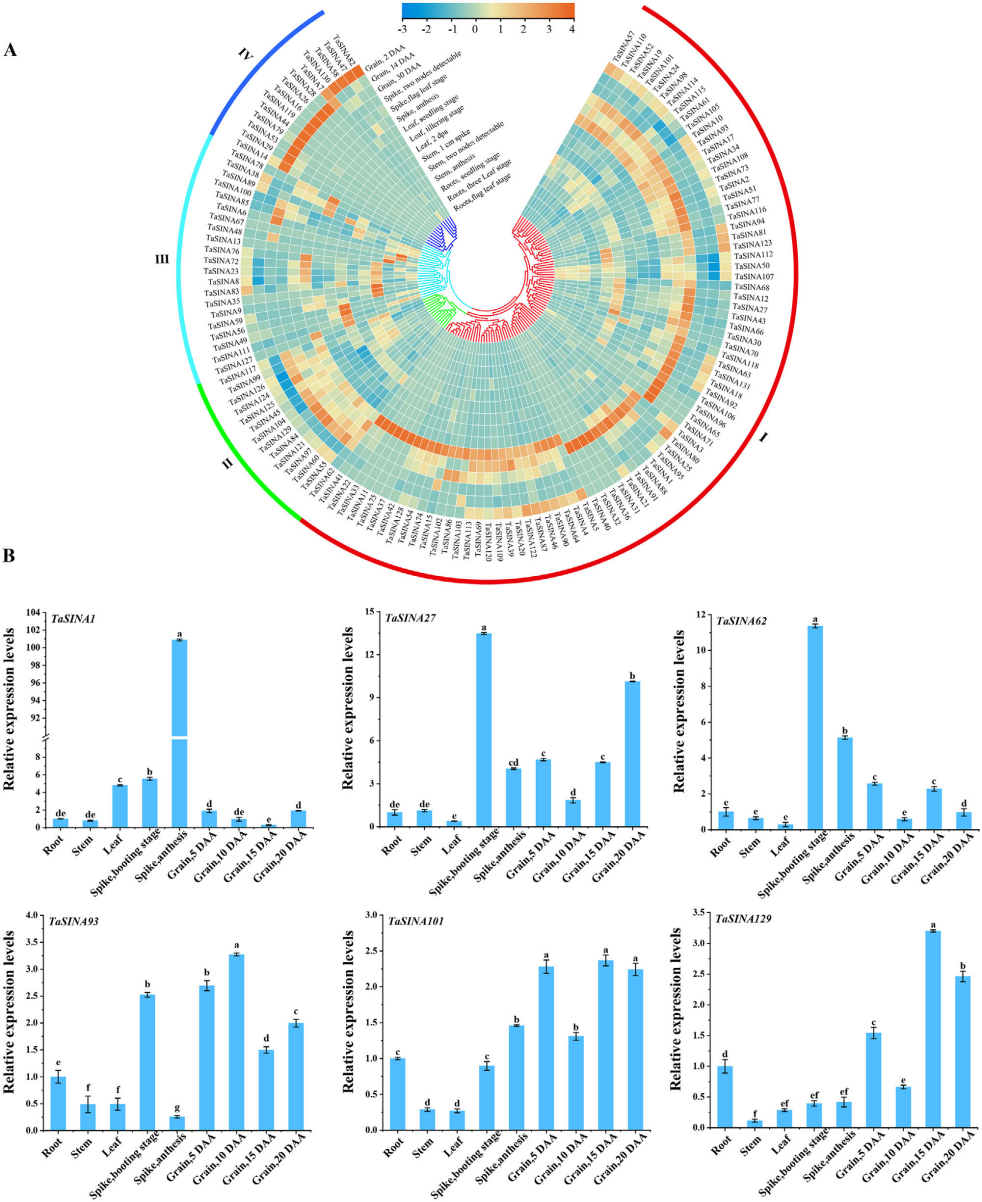
Expression analysis of the *TaSINA* genes. (A) Expression heatmap of *TaSINA* genes in different growth stages and different organs of Chinese Spring. (B) qRT‐PCR analysis of *TaSINA* genes in different organs and developmental periods in wheat. *TaActin* was used as a reference gene. The means were calculated from three biological replicates. The error bars indicate the standard deviation.

To briefly dissect the biological function of TaSINA family members, the expression patterns of *TaSINA* genes in wheat variety Chinese spring in different organs and at different growth stages were analyzed using RNA‐Seq data acquired from the expVIP platform. The results showed that the 131 *TaSINA* genes were classified into four categories, and most of these genes were mainly expressed in the developing spike or grain (Figure 3A). The genes in category I (82/131) were mainly higher expressed in the developing spike compared with other organs, and the genes in category IV (14/131) displayed high expression in the developing grain. The expression of the genes in category II (14/131) showed higher transcript levels in both developing spike and grain. By contrast, only 21 genes in category III showed different expression patterns in diverse organs, for example, *TaSINA13*, *48*, and *67* were mainly expressed in leaves, *TaSINA9*, *35*, and *83* were highly expressed in stems, whereas *TaSINA38*, *49*, *56*, *69*, *78*, and *89* showed high expression level in leaves. This suggested that TaSINA family members could mainly participate in the regulation of spike and grain development.

Six *TaSINA* genes *TaSINA1*, *27*, *62*, *93*, *101*, and *129*, which are highly expressed in developing spikes or grains were selected to verify their expression level with qRT‐PCR assay. The qRT‐PCR results revealed that these genes displayed different expression patterns in diverse organs but were still highly expressed in the developing spike or grain (Figure 3B). This indicated that *TaSINA* genes could play vital roles in the spike and grain development in wheat.

### 3.3 The genomic polymorphism of *TaSINA* family genes and their relationship with grain‐related traits

For further studying the biological function of *TaSINA* family genes in wheat, the genomic polymorphism was analyzed using 681 wheat accessions in population I, which genomic re‐sequencing data acquired from the WheatUnion platform. To ensure broad representativeness, the haplotype possessed in <100 wheat accessions in population I was not considered. The genomic polymorphism analysis displayed that 46.56% of the *TaSINA* family genes (61/131) had two or three haplotypes, only one gene had more than three haplotypes, and 52.67% of the genes (69/131) did not display different haplotypes (Table S7). The distribution frequency of haplotypes in different *TaSINA* family subclades showed that subclades II, V, and VI contained a large proportion of genes (>50%) with different haplotypes (Figure S1).

**FIGURE 4:**
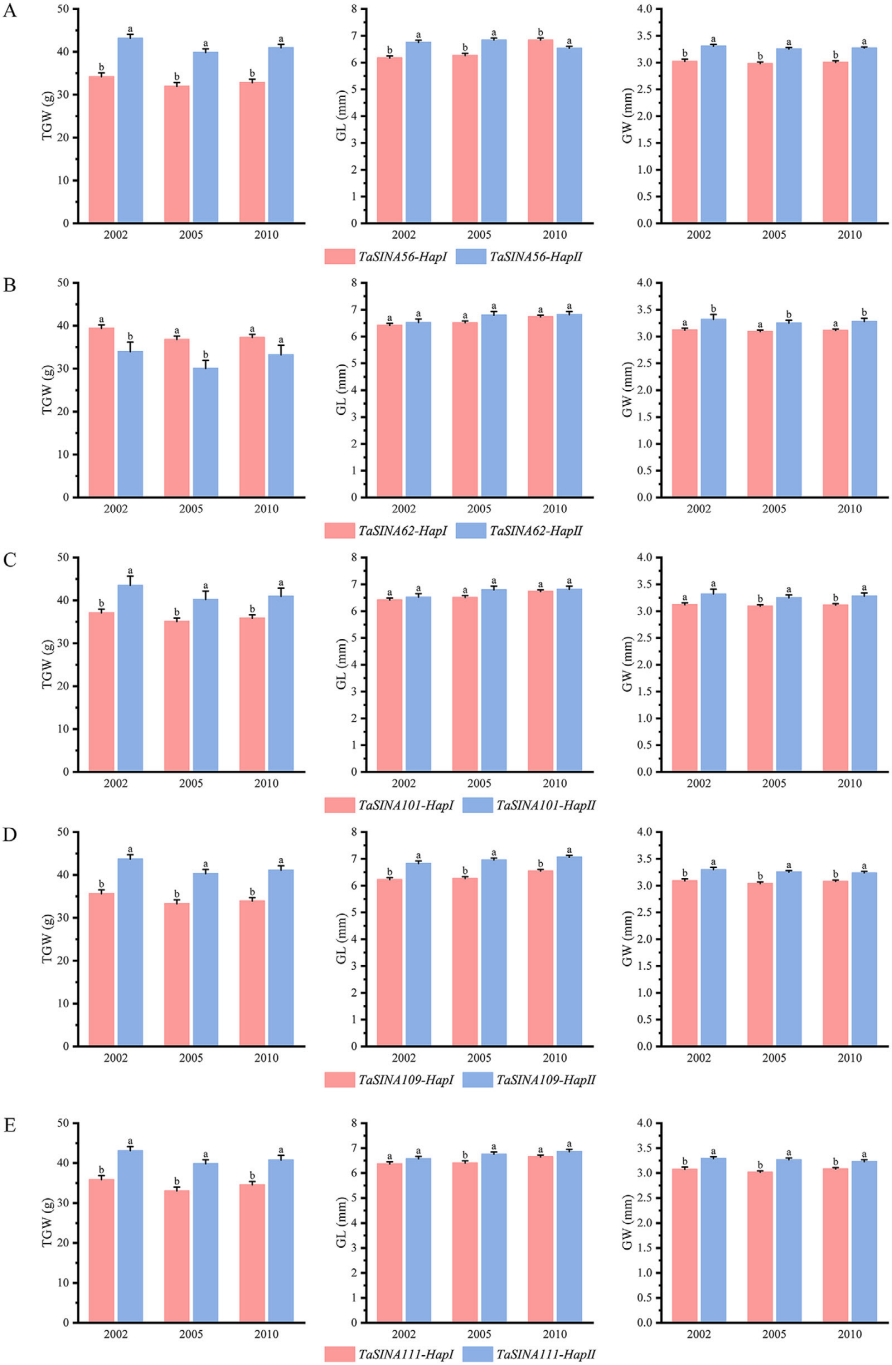
Association analysis between grain‐related traits (in 3 years) and different haplotypes of five *TaSINA* genes. Significant associations were identified between 1000‐grain weight (TGW), grain length (GL), and grain width (GW) with different *TaSINA* haplotypes, including *TaSINA56* (A), *TaSINA62* (B), *TaSINA101* (C), *TaSINA109* (D), and *TaSINA111* (E). TGW, GL, and GW mean TGW, GL, and GW, respectively. The error bars indicate the standard error. The different letters denote a significant difference between means (*p* < 0.05).

Since the transcript expression pattern of *TaSINA* genes indicated their roles in grain development, the relationship between grain weight and haplotypes of *TaSINA* genes was analyzed. The 62 *TaSINA* genes that possessed different haplotypes were used to perform association analysis with grain‐related traits from 122 wheat accessions in population II with data acquired from a previous study. Among them, five genes, naming *TaSINA56*, *62*, *101*, *109*, and *111*, had a significant correlation (*p* < 0.05) with TGW in 3 years (Figure 4; Table S8). The varieties with allele *TaSINA56‐Hap II* exhibited significantly higher TGW and GW compared to those with allele *TaSINA56‐Hap I* in 3 years (Figure 4A). However, *TaSINA56‐Hap II* allele showed a higher GL only in 2002 and 2005, and a lower GL in 2010 (Figure 4A). The varieties with allele *TaSINA62‐Hap I* showed significantly higher TGW compared to *TaSINA62‐Hap II*, but with a lower GW, in 3 years (Figure 4B). The varieties with allele *TaSINA101‐Hap II* showed significantly higher TGW and GW compared with *TaSINA101‐Hap I* in 3 years (Figure 4C). However, no differences were observed between GL with two haplotypes in *TaSINA62* or *101* (Figure 4B,C). The varieties with allele *TaSINA109‐Hap II* and *TaSINA111‐Hap II* both exhibited significantly higher TGW and GW in 3 years (Figure 4D,E). The significant association between GL with two haplotypes of *TaSINA109‐Hap II* and *TaSINA111‐Hap II* was observed in three and 2 years, respectively (Figure 4D,E). The results suggested that these five genes could play a role in the development of wheat grains, and verification with a new natural population containing more wheat accessions should be further performed.

### 3.4 KASP marker development and elite haplotype analysis of *TaSINA101* and *TaSINA109*

**FIGURE 5:**
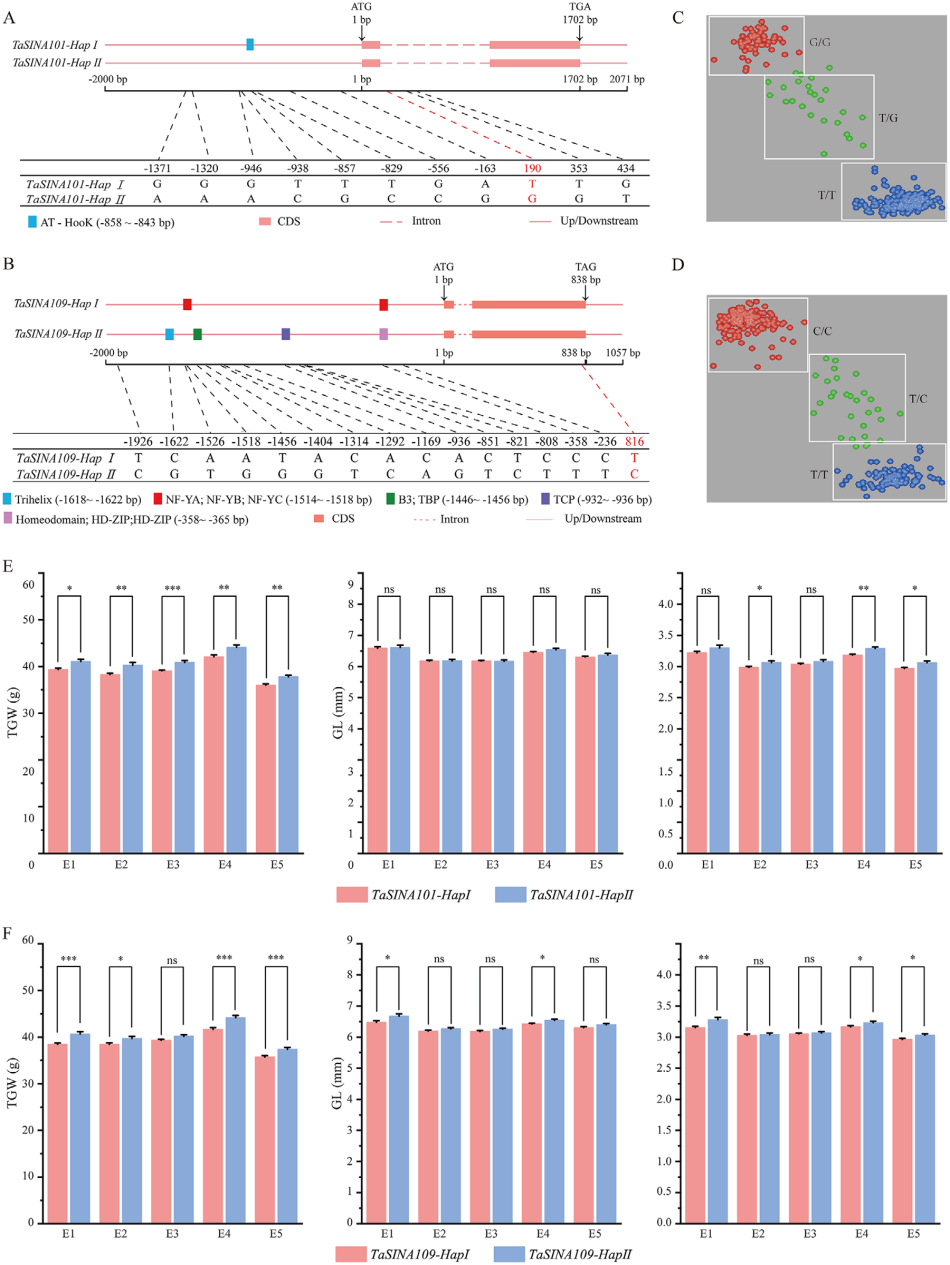
The kompetitive allele‐specific PCR (KASP) marker development and association analysis of *TaSINA101* and *TaSINA109*. Schematic diagram of gene structure and SNPs existed in *TaSINA101* (A) and *TaSINA109* (B) haplotypes. Exons were represented by red boxes, introns were denoted by red dotted lines, and the different *cis*‐elements that existed in the promoter of different haplotypes were represented by different color boxes. The molecular markers were developed based on the polymorphic SNP sites (red color) at position 190 bp away from the ATG of *TaSINA101* and 816 bp away from the ATG of *TaSINA101*, respectively. A total of 292 wheat accessions were divided into different haplotypes validated with the TaSINA101‐KASP (C) and TaSINA109‐KASP (D) marker, respectively. Association analysis of haplotypes of *TaSINA101* (E) and *TaSINA109* (F) with 1000‐grain weight (TGW), grain width (GW), and grain length (GL) in five environments. The error bars indicate the standard error. The different letters denote a significant difference between means (*p* < 0.05).

To verify these association analysis results, the grain weight and size of 292 wheat accessions in population III grown in different regions (five environments) were used to verify the relationship between the haplotypes of these five genes and grain‐related traits. The KASP markers were successfully developed only in *TaSINA101* and *TaSINA109* genes. According to the genomic re‐sequencing data of the WheatUnion platform, at least 11 and 16 SNP sites were identified in the genomic sequences of *TaSINA101* and *TaSINA109*, respectively (Figure 5A,B). Among them, the SNP polymorphism site at 190 bp away from the initiation codon of the *TaSINA101* gene and at 816 bp away from that of the *TaSINA109* gene, were applied to develop KASP markers, respectively (Figure 5A,B). The 292 wheat accessions were finally divided into different haplotypes validated with the KASP marker (Figure 5C,D; Tables S9 and S10). Significant association (*p* < 0.05) between two *TaSINA101* haplotypes with TGW, GL, and GW were observed in five, zero, and three environments, respectively (Figure 5E). The varieties with allele *TaSINA101‐Hap II* had significantly higher grain weight and width than those with *TaSINA101‐Hap I*. Similarly, two *TaSINA109* haplotypes were significantly associated (*p* < 0.05) with TGW, GL, and GW in four, two, and three environments, respectively (Figure 5F). The varieties with allele *TaSINA109‐Hap II* had significantly higher grain weight and size than those with *TaSINA109‐Hap I*. This suggested that *TaSINA101‐Hap II* and *TaSINA109‐Hap II* could be elite allelic variations for improving grain traits in wheat.

### 3.5 Specific expression and location of TaSINA101 in wheat

**FIGURE 6:**
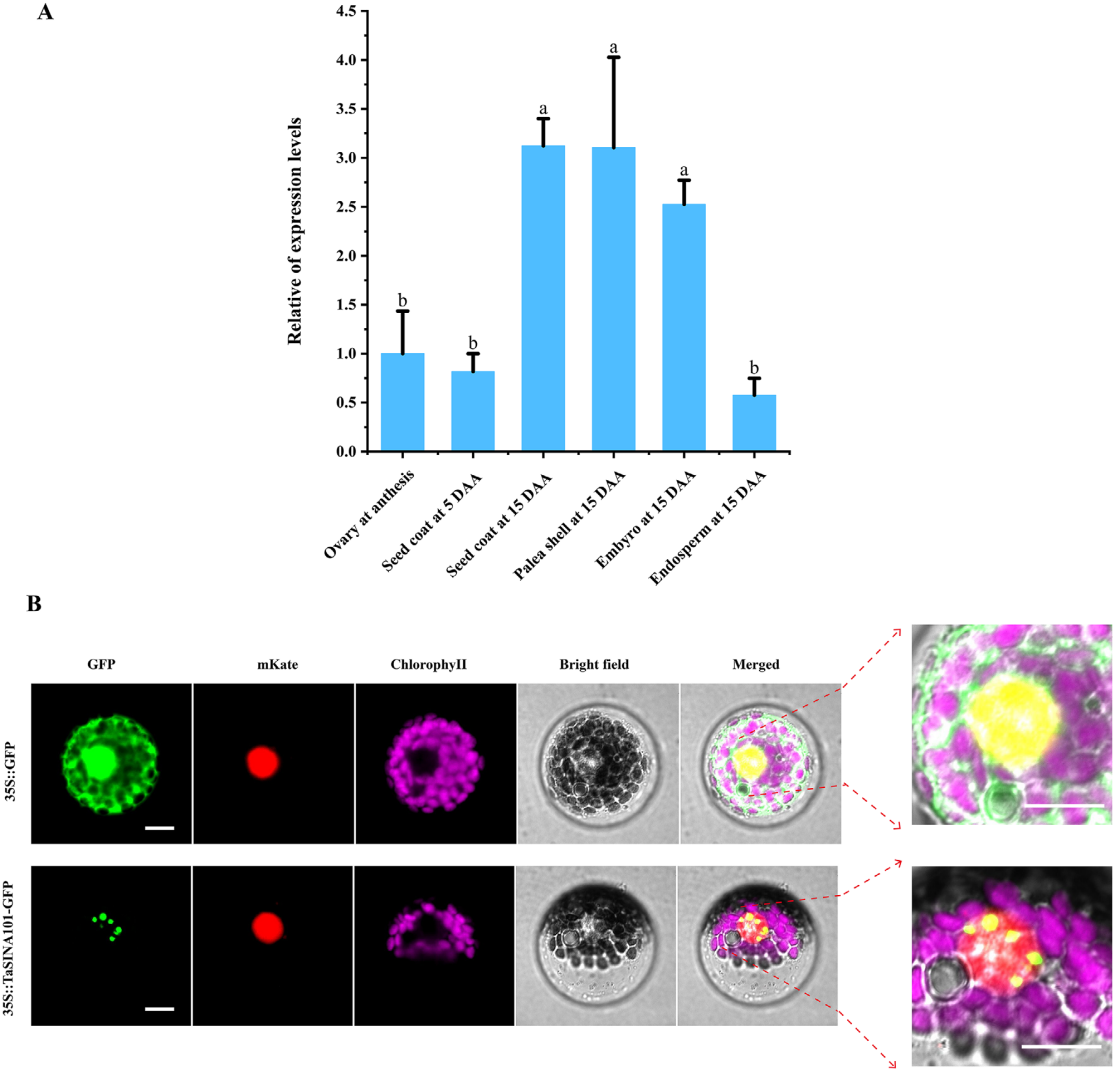
The expression of *TaSINA101* in wheat grain and the subcellular localization of TaSINA101 in wheat protoplast. (A) qRT‐PCR analysis of *TaSINA101* transcripts in the different portions of wheat grain. DAA, days after anthesis. *TaActin* was used as a reference gene. The means were calculated from three biological replicates. The error bars indicate the standard deviation. The different letters denote a significant difference between means (*p* < 0.05). (B) Localization of TaSINA101 by transiently expressing TaSINA101‐YFP in the protoplast of wheat. The nuclear localization signal (NLS) fused with monomeric far‐red fluorescent protein mKate was used as the nucleus marker. Bar = 10 µm.

Since the significant association between two *TaSINA101* haplotypes with TGW was observed in five environments (Figure 5E), the *TaSINA101* gene was then selected to future analyze its biological function. The qRT‐PCR results showed that the *TaSINA101* gene displayed significantly highest expression levels in developing grains compared with that in other organs in wheat (Figure 3B), therefore the specific expression position of the *TaSINA101* gene in wheat grain was further dissected. The qRT‐PCR assay revealed that the *TaSINA101* gene was highly expressed at the palea shell, seed coat, and embryo in the 15‐day grain after anthesis (Figure 6A).

To investigate the spatial localization of TaSINA101 protein in wheat cells, the pro35S::TaSINA101‐eYFP vector was introduced into wheat protoplasts, and the pro35S::eYFP vector was used as a control. The localization of TaSINA101‐eYFP signals displayed that TaSINA101 protein unevenly localized in the nucleus (Figure 6B). The NLS was fused with far‐red fluorescent reporter protein mKATE to confirm the nucleus location of TaSINA101.

### 3.6 Heterologous expression analysis of *TaSINA101* genes in rice

**FIGURE 7:**
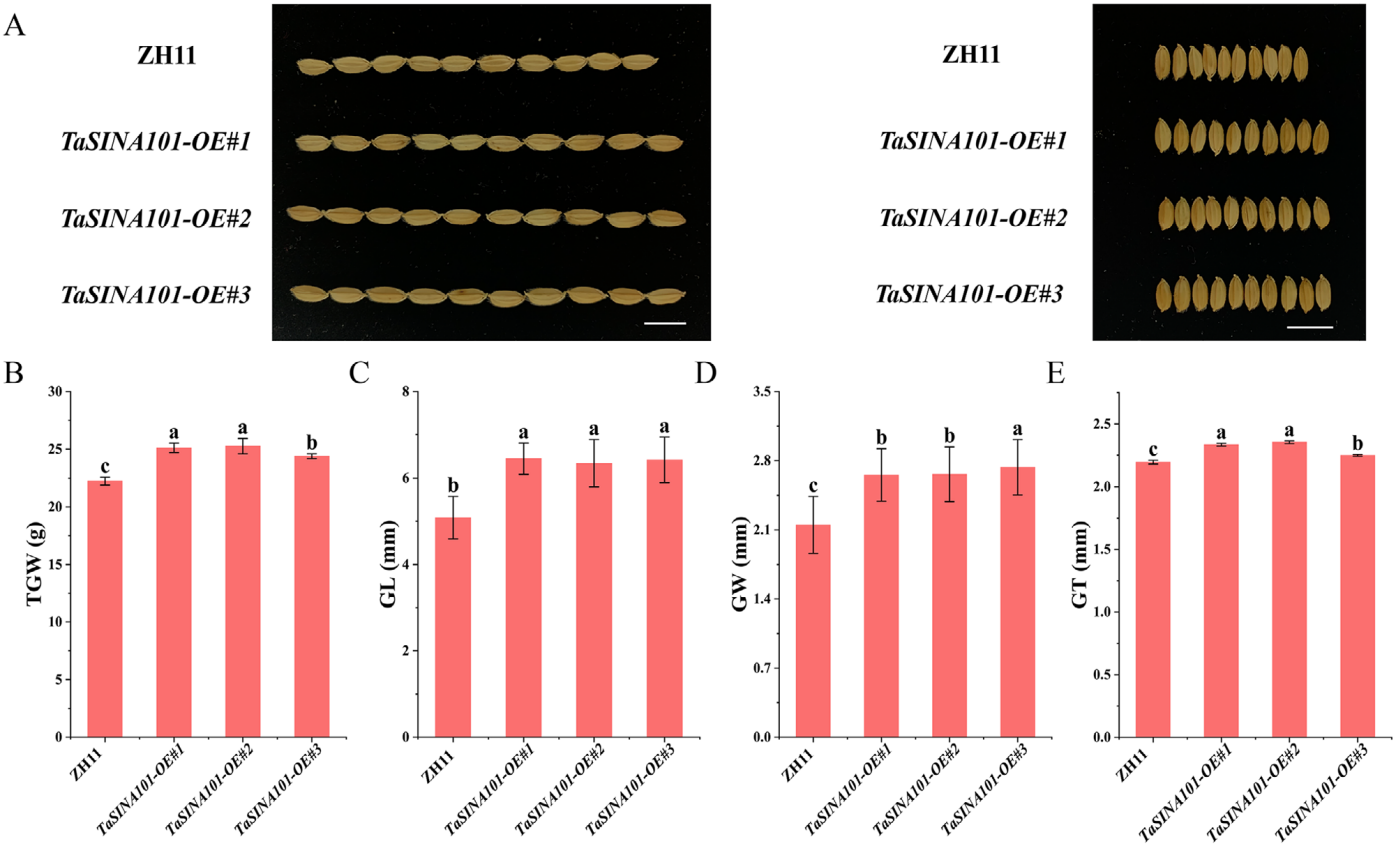
The morphology, weight, and size of rice grain in *TaSINA101* overexpression lines and wild type. (A) The grain morphologies of wild type ZH11 and three *TaSINA101‐OE* lines in rice. Bar = 1 cm. The 1000‐grain weight (TGW) (B), grain length (GL) (C), and grain width (GW) (D) of wild‐type ZH11 and three *TaSINA101‐OE* lines in rice. At least 200 seeds were examined in three *TaSINA101‐OE* lines and their wild type ZH11, with three biological repeats. The error bars represent the standard deviation. The different letters denote a significant difference between means (*p* < 0.05). GT, grain thickness.

To further confirm whether the *TaSINA101* gene was involved in the regulation of wheat grain development, the coding region of *TaSINA101* was constructed with a 35S promoter and then transformed into rice cultivar ZH11. Three homozygous *TaSINA101* transgenic lines *TaSINA101‐OE #1*, *#2*, and *#3* were used to observe the grain‐related traits in rice, and they displayed higher grain weight, length, width, and thickness compared with wild type ZH11 (Figure 7A–E). There was no significant morphological difference between three *TaSINA101‐OE* lines and wild‐type plants in mature stage (Figure S2). These results showed that *SINA* family genes, at least *TaSINA101*, could serve a critical function in grain development in wheat.

## 4 DISCUSSION

Recently, the constantly emerging genomic data have promoted the understanding of pangenome and functional genomic research in plants (W. Guo et al., 2020; Wang et al., 2020). Based on the publication of a high‐quality Wheat Spring Reference genome (IWGSC), the genome sequences assembling of multiple hexaploid wheat varieties, a mass of high‐quality wheat resequencing data, and abundant published phenotypic data of agronomic traits in diverse wheat varieties, a new approach to identify elite alleles in wheat is provided, which may improve breeding accuracy and efficiency (Athiyannan et al., 2022; Cheng et al., 2019; W. Guo et al., 2020; Hao et al., 2020; Walkowiak et al., 2020; Z. Yang et al., 2022; Y. Zhou et al., 2020). In this study, the published resequencing data and phenotypic data in wheat were combined to identify and evaluate haplotypes of *TaSINA* family genes. The relationship between different haplotypes and grain‐related traits was verified with *TaSINA101* and *TaSINA109* genes. Our study revealed that the integration of diverse databases provided convenient conditions for the study of gene function, the development of molecular functional markers, and the design of molecular‐assisted breeding.

TGW, as one of the pivotal factors affecting wheat yield, has been significantly improved over the evolution course from diploid to tetraploid and then to widely planted hexaploid bread wheat, which may be due to the accumulation of superior haplotypes associated with TGW (L. Guo et al., 2022; Y. Wang et al., 2019). So far, abundant elite haplotypes have been found to associate with higher TGW in wheat, such as TASUS2‐2A‐HAPA, TAGS5‐3A‐T, TaAGP‐S1‐7A‐HAPI, TABT‐6B‐HAP1, TANAC019‐B1‐HAP‐2, Tada1‐A‐HAPI, TaNRT 2L12‐HAP‐6B‐1, TasDIR1‐haP‐4A‐2, and Tacyp78A5‐AP‐hapi (L. Guo et al., 2022; Hou et al., 2014, 2017; Huang et al., 2020; H. Liu et al., 2020; Liu et al., 2020; L. Ma et al., 2016; Y. Wang et al., 2019; Wang et al., 2020). In this study, the relationship of 62 *TaSINA* family genes that possessed different haplotypes with grain traits was assessed. The results showed that significant differences existed in TGW, GL, and GW (3‐year data) between different haplotypes of five genes. Bread wheat has experienced asymmetric selection in the A, B, and D sub‐genomes. Compared with the D sub‐genome, more genes and genomic regions in the A and B sub‐genomes have been identified as the target for breed improvement (Hao et al., 2020). Intriguingly, the *TaSINA* family genes, which possessed different haplotypes were mainly distributed in A and B sub‐genomes (85.48%, 53/62) (Table S7). Many elite crop alleles affecting important agronomic traits have been applied in the breeding process (Bhat et al., 2021). For example, through large‐scale whole‐genome resequencing and haplotype analysis, elite haplotypes in rice and pigeon pea have been obtained during the breeding program, and these elite haplotypes have been widely used in breeding selection (Abbai et al., 2019; Sinha et al., 2020). Therefore, the elite haplotypes of five *TaSINA* genes are useful genetic resources for wheat molecular marker‐assisted breeding in the future.

Most of the *TaSINA* family genes were generally highly expressed in developing spike or grain, indicating these genes could participate in the regulation of wheat spike and grain development. The haplotypes of *TaSINA56*, *TaSINA62*, *TaSINA101*, *TaSINA109*, and *TaSINA111* were significantly correlated with grain‐related traits, further indicating their roles in grain development, ultimately affecting yield‐related traits in wheat. It has been reported that overexpression of *TaDISI*, named *TaSINA62* in this study, showed a lower germination rate under both drought stress and ABA treatment in transgenic Arabidopsis (Liu et al., 2018). In this study, we found heterologous expression of *TaSINA101* in rice displayed higher grain weight and size compared with wild type. In addition to these five genes, *TaSIRFP‐3A* and *TaSIRFP‐3B*, named *TaSINA129* and *TaSINA97* in this study, respectively, regulate plant growth via regulating sucrose synthase proteins (Park & Jang, 2020). It has also been reported that a TaSINA protein identified in QTL *qYDH.3BL*, named TaSINA100 in this study, promoted root growth, plant biomass, and yield components under various growth conditions (Thomelin et al., 2021). A worldwide distribution analysis indicated that the elite allele of *TaSINA100* was widespread from the 1950s through the CIMMYT wheat breeding program, while only positively selected in Mexico and Australia so far (Thomelin et al., 2021). As a result, *TaSINA* family genes may serve vital roles in wheat development and yield‐related traits.

Functional molecular markers related to agronomic traits, such as grain weight, plant height, photoperiod response, and disease resistance, have been widely used in molecular‐assisted breeding. Several functional molecular markers in diverse genes related to grain traits, such as *TaGW2*, *TaGS5‐3A*, *TaTGW6‐A1*, *TaSDIR1‐4A*, and *TaSus2‐2B*, have been developed in wheat (Hanif et al., 2016; Jiang et al., 2011; L. Ma et al., 2016; Qin et al., 2017; J. Wang et al., 2020). In this study, two KASP functional markers were developed based on polymorphic loci in the mRNA of *TaSINA101* and *TaSINA109*, and association analysis was conducted to verify the potential value in TGW breeding selection. Therefore, the TaSINA101‐KASP and TaSINA109‐KASP markers can be, respectively, used to identify elite haplotypes *TaSINA101‐HAP II and TaSINA10p‐HAP II* alleles during the wheat breeding process.

## 5 CONCLUSIONS

We assessed the roles of *TaSINA* family members in the regulation of grain development by integrating different databases to analyze the expression pattern, genomic polymorphism, and association assay. Two KASP markers were developed to confirm the SNP polymorphism of the *TaSINA101* and *TaSINA109* genes in 292 wheat accessions. The specific expression position in grain and the subcellular localization of TaSINA101 were further analyzed. The phenotypes of *TaSINA101* transgenic lines in rice were also observed to confirm the role of *TaSINA101* in grain development. The findings will provide a strategy for understanding the biological roles of large gene families via integrating different databases, and developed two molecular functional markers, which may be helpful for wheat molecular‐assisted breeding in the future.

## AUTHOR CONTRIBUTIONS

**Tao Chen**: Conceptualization; data curation; funding acquisition; investigation; writing—original draft. **Yongping Miao**: Data curation; formal analysis; investigation. **Fanli Jing**: Formal analysis; investigation. **Weidong Gao**: Investigation. **Yanyan Zhang**: Investigation. **Long Zhang**: Investigation. **Peipei Zhang**: Investigation. **Lijian Guo**: Investigation. **Delong Yang**: Funding acquisition; resources; supervision; writing—review and editing.

## CONFLICT OF INTEREST STATEMENT

The authors declare no conflicts of interest.

## Supporting information


**Figure S1**. The frequency of different haplotypes in *TaSINA* genes of different *TaSINA* family subclades. The number of haplotypes of the *TaSINA* family genes is represented in different colors.
**Figure S2**. The morphology of *TaSINA101* overexpression lines and wild type ZH11 in the mature stage. Bar = 10 cm.

**Table S1**. Wheat accessions in Population I which genomic information acquired from the WheatUnion platform.
**Table S2**. Wheat accessions in Population II which grain traits data acquired from previous study.
**Table S3**. Wheat accessions in Population III which were grown in five environments.
**Table S4**. Primer sequences used in this study.
**Table S5**. Transcriptome data of *TaSINA* family genes in different organs.
**Table S6**. Characteristics of *TaSINA* family members.
**Table S7**. Number of haplotypes of *TaSINA* family members.
**Table S8**. Association analysis of TGW, GL and GW with different haplotypes of five *TaSINA* genes.
**Table S9**. Nucleotide polymorphisms detected in *TaSINA101* gene.
**Table S10**. Nucleotide polymorphisms detected in *TaSINA109* gene.

## Data Availability

The datasets used and/or analyzed during the current study are available from the corresponding author on reasonable request. Some of the data are shown in Supporting Information.
