# Supplementary material for: Genomic‐wide analysis reveals seven in absentia genes regulating grain development in wheat (Triticum aestivum L.)
Source: Plant Genome. 2024 Jun 5;17(3):e20480. doi: 10.1002/tpg2.20480 (PMC12807368; doi:10.1002/tpg2.20480)
Supplement: Supplementary file 1 [file TPG2-17-e20480-s001.docx]

**Table S1 Wheat accessions in Population I which genomic information acquired from the WheatUnion platform**

| **No.** | **ID** | **No.** | **ID** | **No.** | **ID** | **No.** | **ID** |
| --- | --- | --- | --- | --- | --- | --- | --- |
| 1 | Zang1817 | 172 | ZWT | 343 | S25 | 514 | S225 |
| 2 | TW001 | 173 | ZYJWLQ | 344 | S26 | 515 | S227 |
| 3 | TW002 | 174 | MP001 | 345 | S27 | 516 | S228 |
| 4 | TW003 | 175 | MP002 | 346 | S28 | 517 | S229 |
| 5 | TW004 | 176 | MP003 | 347 | S29 | 518 | S230 |
| 6 | TW005 | 177 | MP004 | 348 | S30 | 519 | S231 |
| 7 | TW006 | 178 | MP005 | 349 | S31 | 520 | S232 |
| 8 | TW007 | 179 | MP006 | 350 | S32 | 521 | S233 |
| 9 | TW008 | 180 | MP007 | 351 | S33 | 522 | S234 |
| 10 | TW009 | 181 | MP008 | 352 | S34 | 523 | S235 |
| 11 | TW010 | 182 | MP009 | 353 | S35 | 524 | S236 |
| 12 | TW011 | 183 | MP010 | 354 | S36 | 525 | S237 |
| 13 | TW012 | 184 | MP011 | 355 | S37 | 526 | S238 |
| 14 | TW013 | 185 | MP012 | 356 | S38 | 527 | S239 |
| 15 | TW014 | 186 | MP013 | 357 | S39 | 528 | S240 |
| 16 | TW015 | 187 | MP014 | 358 | S40 | 529 | S241 |
| 17 | TW016 | 188 | MP015 | 359 | S41 | 530 | S242 |
| 18 | TW017 | 189 | MP016 | 360 | S42 | 531 | S243 |
| 19 | TW018 | 190 | MP017 | 361 | S43 | 532 | S244 |
| 20 | TW019 | 191 | MP018 | 362 | S44 | 533 | S245 |
| 21 | TW020 | 192 | MP019 | 363 | S45 | 534 | S246 |
| 22 | TW021 | 193 | MP020 | 364 | S46 | 535 | S247 |
| 23 | TW022 | 194 | MP021 | 365 | S47 | 536 | S248 |
| 24 | TW023 | 195 | MP022 | 366 | S48 | 537 | S249 |
| 25 | TW024 | 196 | MP023 | 367 | S49 | 538 | S250 |
| 26 | TW025 | 197 | MP024 | 368 | S50 | 539 | S251 |
| 27 | TW026 | 198 | MP025 | 369 | S51 | 540 | S252 |
| 28 | TW027 | 199 | MP026 | 370 | S52 | 541 | S253 |
| 29 | TW028 | 200 | MP027 | 371 | S56 | 542 | S254 |
| 30 | TW029 | 201 | MP028 | 372 | S57 | 543 | S255 |
| 31 | TW030 | 202 | MP029 | 373 | S58 | 544 | S256 |
| 32 | TW031 | 203 | MP030 | 374 | S59 | 545 | S257 |
| 33 | TW032 | 204 | MP031 | 375 | S60 | 546 | S258 |
| 34 | TW033 | 205 | MP032 | 376 | S61 | 547 | S259 |
| 35 | TW034 | 206 | MP033 | 377 | S62 | 548 | S260 |
| 36 | TW035 | 207 | MP034 | 378 | S64 | 549 | S261 |
| 37 | TW036 | 208 | MP035 | 379 | S67 | 550 | S262 |
| 38 | TW037 | 209 | MP036 | 380 | S72 | 551 | S264 |
| 39 | TW038 | 210 | MP037 | 381 | S73 | 552 | S265 |
| 40 | TW039 | 211 | MP038 | 382 | S74 | 553 | S266 |
| 41 | TW040 | 212 | MP039 | 383 | S75 | 554 | S267 |
| 42 | TW041 | 213 | MP040 | 384 | S76 | 555 | S268 |
| 43 | TW043 | 214 | MP041 | 385 | S77 | 556 | S270 |
| 44 | TW044 | 215 | MP042 | 386 | S78 | 557 | S271 |
| 45 | TW045 | 216 | MP043 | 387 | S79 | 558 | S272 |
| 46 | TW046 | 217 | MP044 | 388 | S80 | 559 | S273 |
| 47 | TW047 | 218 | MP045 | 389 | S81 | 560 | S274 |
| 48 | TW048 | 219 | MP046 | 390 | S82 | 561 | S3331 |
| 49 | TW049 | 220 | MP047 | 391 | S83 | 562 | S6554 |
| 50 | TW050 | 221 | MP048 | 392 | S84 | 563 | CS |
| 51 | TW051 | 222 | MP049 | 393 | S86 | 564 | C1 |
| 52 | TW052 | 223 | MP050 | 394 | S87 | 565 | C2 |
| 53 | TW053 | 224 | MP051 | 395 | S88 | 566 | C3 |
| 54 | TW054 | 225 | MP052 | 396 | S89 | 567 | C4 |
| 55 | AFG-L1 | 226 | MP053 | 397 | S90 | 568 | C5 |
| 56 | AFG-L2 | 227 | MP054 | 398 | S91 | 569 | C6 |
| 57 | AFG-L3 | 228 | MP055 | 399 | S92 | 570 | C7 |
| 58 | AMN | 229 | MP056 | 400 | S93 | 571 | C8 |
| 59 | AUT-L1 | 230 | MP057 | 401 | S94 | 572 | C9 |
| 60 | BDHHM | 231 | MP058 | 402 | S95 | 573 | C10 |
| 61 | BIH-L1 | 232 | MP059 | 403 | S96 | 574 | C11 |
| 62 | BJBH | 233 | MP060 | 404 | S97 | 575 | C12 |
| 63 | BMZ | 234 | MP061 | 405 | S98 | 576 | C13 |
| 64 | BQM | 235 | MP062 | 406 | S99 | 577 | C14 |
| 65 | BRA-L1 | 236 | MP063 | 407 | S100 | 578 | C15 |
| 66 | BYM | 237 | MP064 | 408 | S101 | 579 | C16 |
| 67 | CAN-C1 | 238 | MP065 | 409 | S102 | 580 | C17 |
| 68 | CHE-L1 | 239 | MP066 | 410 | S103 | 581 | C18 |
| 69 | CHL-C1 | 240 | MP067 | 411 | S104 | 582 | C19 |
| 70 | CSB | 241 | MP068 | 412 | S105 | 583 | C20 |
| 71 | CYHM | 242 | MP069 | 413 | S106 | 584 | C21 |
| 72 | CZ6406 | 243 | MP070 | 414 | S108 | 585 | C22 |
| 73 | DEU-L1 | 244 | MP071 | 415 | S109 | 586 | C23 |
| 74 | DHM | 245 | MP072 | 416 | S110 | 587 | C24 |
| 75 | DKM | 246 | MP073 | 417 | S111 | 588 | C25 |
| 76 | DXHKYM | 247 | MP074 | 418 | S112 | 589 | C26 |
| 77 | DYH | 248 | MP075 | 419 | S113 | 590 | C27 |
| 78 | ESP-L1 | 249 | MP076 | 420 | S114 | 591 | C28 |
| 79 | FIN-L1 | 250 | MP077 | 421 | S115 | 592 | C29 |
| 80 | FMSY | 251 | MP078 | 422 | S116 | 593 | C30 |
| 81 | FRA-C1 | 252 | MP079 | 423 | S117 | 594 | C31 |
| 82 | GBR-L1 | 253 | MP080 | 424 | S118 | 595 | C32 |
| 83 | GEO-L1 | 254 | MP081 | 425 | S119 | 596 | C33 |
| 84 | GRC-L1 | 255 | MP082 | 426 | S120 | 597 | C34 |
| 85 | GYWLL | 256 | MP083 | 427 | S121 | 598 | C35 |
| 86 | HCM | 257 | MP084 | 428 | S122 | 599 | C36 |
| 87 | HLM | 258 | MP085 | 429 | S123 | 600 | C37 |
| 88 | HMM | 259 | MP086 | 430 | S124 | 601 | C38 |
| 89 | HM | 260 | MP087 | 431 | S125 | 602 | C39 |
| 90 | HNSH | 261 | MP088 | 432 | S126 | 603 | C40 |
| 91 | HRV-L1 | 262 | MP089 | 433 | S127 | 604 | C41 |
| 92 | HTZ | 263 | MP090 | 434 | S128 | 605 | C42 |
| 93 | HUN-L1 | 264 | MP091 | 435 | S129 | 606 | C43 |
| 94 | HZB | 265 | MP092 | 436 | S130 | 607 | C44 |
| 95 | IND-L1 | 266 | MP093 | 437 | S131 | 608 | C45 |
| 96 | IND-L2 | 267 | MP094 | 438 | S132 | 609 | C46 |
| 97 | IRN-L1 | 268 | MP095 | 439 | S133 | 610 | C47 |
| 98 | IRN-L2 | 269 | MP096 | 440 | S134 | 611 | C48 |
| 99 | IRN-L3 | 270 | MP097 | 441 | S135 | 612 | C49 |
| 100 | IRN-L4 | 271 | MP098 | 442 | S136 | 613 | C50 |
| 101 | IRQ-L1 | 272 | MP099 | 443 | S137 | 614 | C51 |
| 102 | IRQ-L2 | 273 | MP100 | 444 | S139 | 615 | C52 |
| 103 | IRQ-L3 | 274 | MP101 | 445 | S140 | 616 | C53 |
| 104 | IRQ-L4 | 275 | MP102 | 446 | S142 | 617 | C54 |
| 105 | ITA-C1 | 276 | MP103 | 447 | S143 | 618 | C55 |
| 106 | ITA-L1 | 277 | MP104 | 448 | S144 | 619 | C56 |
| 107 | ITA-L2 | 278 | MP105 | 449 | S145 | 620 | C57 |
| 108 | JCYLYL | 279 | MP106 | 450 | S146 | 621 | C58 |
| 109 | JiaHM | 280 | MP107 | 451 | S147 | 622 | C59 |
| 110 | JinHM | 281 | MP108 | 452 | S148 | 623 | C60 |
| 111 | JM | 282 | MP109 | 453 | S149 | 624 | C61 |
| 112 | JM8H | 283 | MP110 | 454 | S150 | 625 | C62 |
| 113 | JPN-L1 | 284 | MP111 | 455 | S151 | 626 | ArinaLrFor |
| 114 | KAZ-L1 | 285 | MP112 | 456 | S152 | 627 | CDC_Landmark |
| 115 | KFEH | 286 | MP113 | 457 | S153 | 628 | CDC_Stanley |
| 116 | KFSH | 287 | MP114 | 458 | S154 | 629 | Cadenza |
| 117 | KSBP | 288 | MP115 | 459 | S155 | 630 | Claire |
| 118 | KXSH | 289 | MP116 | 460 | S156 | 631 | Jagger |
| 119 | LGD | 290 | MP117 | 461 | S157 | 632 | Julius |
| 120 | LLX | 291 | MP118 | 462 | S158 | 633 | Lancer |
| 121 | LM | 292 | MP119 | 463 | S159 | 634 | Mace |
| 122 | LTT | 293 | MP120 | 464 | S160 | 635 | Norin61 |
| 123 | LXZXM | 294 | MP121 | 465 | S161 | 636 | Paragon |
| 124 | LZT | 295 | MP122 | 466 | S162 | 637 | Robigus |
| 125 | MEX-L1 | 296 | MP123 | 467 | S163 | 638 | SY_Mattis |
| 126 | MKD-L1 | 297 | MP124 | 468 | S164 | 639 | Spelt |
| 127 | MXM | 298 | MP125 | 469 | S165 | 640 | Weebil |
| 128 | NLD-C1 | 299 | MP126 | 470 | S166 | 641 | Z8425B |
| 129 | NLD-L1 | 300 | MP127 | 471 | S167 | 642 | PH46 |
| 130 | NXWH | 301 | MP128 | 472 | S168 | 643 | PH49 |
| 131 | PAK-L1 | 302 | MP129 | 473 | S169 | 644 | PH51 |
| 132 | PAK-L2 | 303 | MP130 | 474 | S170 | 645 | PH52 |
| 133 | PAK-L3 | 304 | MP131 | 475 | S171 | 646 | PH56 |
| 134 | PRT-L1 | 305 | MP132 | 476 | S172 | 647 | PH68 |
| 135 | PYEQ | 306 | MP133 | 477 | S173 | 648 | PH148 |
| 136 | QCM | 307 | MP134 | 478 | S174 | 649 | PH95 |
| 137 | ROM-L1 | 308 | MP135 | 479 | S175 | 650 | PH105 |
| 138 | RUS-L1 | 309 | MP136 | 480 | S176 | 651 | PH108 |
| 139 | SLZ | 310 | MP137 | 481 | S177 | 652 | PH135 |
| 140 | SM | 311 | MP138 | 482 | S178 | 653 | PH151 |
| 141 | SRB-L1 | 312 | MP139 | 483 | S179 | 654 | PH153 |
| 142 | SRM4H | 313 | MP140 | 484 | S180 | 655 | PH126 |
| 143 | SWE-L1 | 314 | MP141 | 485 | S181 | 656 | PH132 |
| 144 | SYR-L1 | 315 | MP142 | 486 | S182 | 657 | PH133 |
| 145 | SYR-L2 | 316 | MP143 | 487 | S183 | 658 | PH09 |
| 146 | TJBXM | 317 | MP144 | 488 | S184 | 659 | XM01 |
| 147 | TJK-L1 | 318 | MP145 | 489 | S185 | 660 | PH10 |
| 148 | TMM | 319 | S1 | 490 | S186 | 661 | NZ05 |
| 149 | TUR-L1 | 320 | S2 | 491 | S187 | 662 | YM05 |
| 150 | TUR-L2 | 321 | S3 | 492 | S188 | 663 | YM02 |
| 151 | TUR-L3 | 322 | S4 | 493 | S203 | 664 | YM07 |
| 152 | TUR-L4 | 323 | S5 | 494 | S204 | 665 | YM03 |
| 153 | UKR-L1 | 324 | S6 | 495 | S205 | 666 | NZ10 |
| 154 | UKR-L2 | 325 | S7 | 496 | S206 | 667 | S141 |
| 155 | URY-L1 | 326 | S8 | 497 | S207 | 668 | AS360 |
| 156 | URY-L2 | 327 | S9 | 498 | S208 | 669 | AS362 |
| 157 | USA-C1 | 328 | S10 | 499 | S209 | 670 | 18C429 |
| 158 | UZB-C1 | 329 | S11 | 500 | S210 | 671 | 18C430 |
| 159 | UZB-L1 | 330 | S12 | 501 | S211 | 672 | 18C448 |
| 160 | WYM | 331 | S13 | 502 | S212 | 673 | 18C450 |
| 161 | XFS | 332 | S14 | 503 | S213 | 674 | 3987 |
| 162 | XHP | 333 | S15 | 504 | S214 | 675 | 3989 |
| 163 | XYLH | 334 | S16 | 505 | S215 | 676 | 3970 |
| 164 | YASY | 335 | S17 | 506 | S216 | 677 | AS356 |
| 165 | YMBF | 336 | S18 | 507 | S217 | 678 | XD2 |
| 166 | YM | 337 | S19 | 508 | S218 | 679 | XLM2 |
| 167 | YNSW | 338 | S20 | 509 | S219 | 680 | XS2 |
| 168 | YZM | 339 | S21 | 510 | S220 | 681 | XZ892 |
| 169 | ZDSH | 340 | S22 | 511 | S221 |  |  |
| 170 | ZJH | 341 | S23 | 512 | S223 |  |  |
| 171 | ZP | 342 | S24 | 513 | S224 |  |  |

**Table S2. Wheat accessions in Population II which grain traits data acquired from previous study**

| **No.** | **Accession** | **No.** | **Accession** |
| --- | --- | --- | --- |
| 1 | AiFeng3Hao | 62 | Lanxizaoxiaomai |
| 2 | DF_SDBaiBianSui | 63 | DF_SDLaoLaiXia |
| 3 | DF_GSBaiDaTou | 64 | Laomai |
| 4 | BaiHuaMai | 65 | Laotutou |
| 5 | DF_SXBaiHuoMai | 66 | DF_QHLiuYueHuang |
| 6 | DF_SCBaiMangXiaoMai | 67 | Liuzhutou |
| 7 | BaiMaZha | 68 | Louguding |
| 8 | BoNong3217 | 69 | Mangxiaomai |
| 9 | Baiqiumai | 70 | MaZhaMai |
| 10 | Baiyoumai | 71 | MianYang11 |
| 11 | Bejing8 | 72 | MianYang26Hao |
| 12 | Bendihuanghuamai | 73 | XZD_MoZongZhuoGa |
| 13 | XZD_BianBaChunMai6 | 74 | Mentana |
| 14 | BiMa1Hao | 75 | NingChun4Hao |
| 15 | BiMa4Hao | 76 | Orofen |
| 16 | ChanBuZi | 77 | PaoZiMai |
| 17 | YCL_ChangZhi6406 | 78 | Pingyang27 |
| 18 | ChengDouGuangTou | 79 | Qiangchangmai |
| 19 | ZhongGuoChun | 80 | YC_XZRiKaZe54 |
| 20 | Akagomughi | 81 | YC_XZRiKaZe8 |
| 21 | Congyanghongmai | 82 | DF_NXShanMai |
| 22 | DF_SHXChunXiaoMai | 83 | ShanNong7859 |
| 23 | Chushanbao | 84 | DanJiaZhuang54 |
| 24 | Dahongmai | 85 | Shuilizhan |
| 25 | Dakoumai | 86 | SuMai3Hao |
| 26 | DF_SDDaLiBanMang | 87 | TaiShan1Hao |
| 27 | Dayuhua | 88 | Tongjiabaxiaomai |
| 28 | Dianxihongkeyangmai | 89 | Tumangmai |
| 29 | DF_SXDingXingZhai | 90 | WangShuiBai |
| 30 | YCL_EnMai4 | 91 | WenMai6Hao |
| 31 | Fan6 | 92 | XZD_WuJiangZhuo |
| 32 | FengChan3Hao | 93 | Liyuanmai |
| 33 | Fengkang2 | 94 | Xiaofoshou |
| 34 | Fengmai11 | 95 | Xiaohongpi |
| 35 | FuMai | 96 | DF_SHXXiaoSanYueH |
| 36 | Funo | 97 | XiaoYan6Hao |
| 37 | GanMai8Hao | 98 | XinKeHan9Hao |
| 38 | CI 12203 | 99 | XiNong6028 |
| 39 | GaoYou503 | 100 | DF_SDXiShanBianSui |
| 40 | Gaoyuan506 | 101 | Yanan11 |
| 41 | DF_QHGeJiaXiang | 102 | YC_YanNong15 |
| 42 | Hanzhongbai | 103 | DF_GSYiZhiMai |
| 43 | DF_XJHongJinBaoYin | 104 | YouZiMai |
| 44 | Huomai | 105 | YuMai18 |
| 45 | Hongtuzi | 106 | YuMai2Hao |
| 46 | Huining10 | 107 | YuMai7Hao |
| 47 | Huoliaomai | 108 | YC_XZZangDong4 |
| 48 | HuoLiYan | 109 | Zaowutian |
| 49 | Hongmangmai | 110 | ZaoXiaoMai |
| 50 | Jiahongmai | 111 | XZD_ZhaHong |
| 51 | JiangDongMen | 112 | LuMai1Hao |
| 52 | Jichun1016 | 113 | ChuanMai22 |
| 53 | JiMai20 | 114 | ZhengMai9023 |
| 54 | JiNan2Hao | 115 | ZhongYou9507 |
| 55 | JiNan9Hao | 116 | YCL_JiNan17 |
| 56 | Jiangmai | 117 | YCL_YanZhan1 |
| 57 | DF_SCKangDing1 | 118 | NeiXiang5Hao |
| 58 | Kangxiu10 | 119 | LR_China2 |
| 59 | Kashibaipi | 120 | ZhuShiMai |
| 60 | Kefeng3 | 121 | ZiJieHong |
| 61 | DF_GSLanHuaMai | 122 | Zipi |

**Table S3. Wheat accessions in Population III which were grown in five environments**

| **No.** | **Accession** | **No.** | **Accession** | **No.** | **Accession** |  |
| --- | --- | --- | --- | --- | --- | --- |
|  |  |  |  |  |  |  |
| 1 | 09-228-9-2-1-1-1-2 | 101 | Lantian35 | 201 | 10-160-6-1-1-5-2-3 |  |
| 2 | 11-257-4--1 | 102 | Lantian4 | 202 | 1389-2 |  |
| 3 | 12-37-10-1-4 | 103 | Lanxuan1 | 203 | 9840-0-3-2 |  |
| 4 | 1R1 | 104 | Linfeng3 | 204 | 988-4-2-4-1 |  |
| 5 | 1R14 | 105 | Linhan234 | 205 | 99384-2-1 |  |
| 6 | 1R25 | 106 | Longjian103 | 206 | A80-3-1-1-3 |  |
| 7 | 21-30 | 107 | Longjian107 | 207 | C130-5-1-1 |  |
| 8 | 22-23 | 108 | Longjian108 | 208 | C42-2-3-3 |  |
| 9 | 27-1 | 109 | Longjian110 | 209 | C49-1-1-1-2 |  |
| 10 | 29-2-2 | 110 | Longjian127 | 210 | C78-3-6-1-3 |  |
| 11 | 85-173-4 | 111 | Longjian386 | 211 | Fanmai8 |  |
| 12 | 94164-1 | 112 | Longmai079 | 212 | Huaimai18 |  |
| 13 | A8-4-4-2 | 113 | Longmai838 | 213 | Jimai20 |  |
| 14 | B11-2-3-1-1-2 | 114 | Longmai844 | 214 | Jimai262 |  |
| 15 | B160-2-2-1-1 | 115 | Longyu218 | 215 | Jimai4 |  |
| 16 | B17-2-3-2-1 | 116 | Longyuan235 | 216 | Jinmai17 |  |
| 17 | B61-2-3-2-1 | 117 | Longyuan937 | 217 | Jingdong 82 Dong 307 |  |
| 18 | C14-14-1 | 118 | Longyuan961 | 218 | Jinghua1 |  |
| 19 | C47-1-1-2 | 119 | Longyuan036 | 219 | Jingshuang2hao |  |
| 20 | C53-3-2-1-1 | 120 | Longzhong1hao | 220 | Lantain14 |  |
| 21 | C72-6-1-2-1 | 121 | Longzimai1 | 221 | Lantian15 |  |
| 22 | Dd52-17-3-2 | 122 | Lumai14 | 222 | Lin138 |  |
| 23 | E69-4-1 | 123 | Lumai3 | 223 | Linhan538 |  |
| 24 | E71-2-6 | 124 | Lunxuan987 | 224 | Longjian111 |  |
| 25 | E72-2-2 | 125 | Luohan3 | 225 | Longjian294 |  |
| 26 | H-4-2-2-1 | 126 | Luohan8 | 226 | Longmai847 |  |
| 27 | Q9086 | 127 | Luohan9hao | 227 | Longyu5hao |  |
| 28 | Baiqimai | 128 | Luoyou7 | 228 | Longzhong2hao |  |
| 29 | Bainong160 | 129 | Ningmai12hao | 229 | Lumai17 |  |
| 30 | Beijing837 | 130 | Ningmai5 | 230 | Lumai5 |  |
| 31 | Beijing 8686 | 131 | Nongda135 | 231 | Luohan11 |  |
| 32 | Xifeng24 | 132 | Pingliang35 | 232 | Luohan2 |  |
| 33 | Beinong2hao | 133 | Qinmai3 | 233 | Luohan6 |  |
| 34 | Cangmai6001 | 134 | Qingmai7 | 234 | Luohan7 |  |
| 35 | Changle5hao | 135 | Qingnong4 | 235 | Luomai22 |  |
| 36 | Dongnong024 | 136 | Qingshan851 | 236 | Luomai23 |  |
| 37 | Dong03-07 | 137 | Qingshan782 | 237 | Luomai8 |  |
| 38 | Fengchan1 | 138 | Qingshan843 | 238 | Qingnong3 |  |
| 39 | Fuzhaung30 | 139 | Shannongyoumai2 | 239 | Shannongfu63 |  |
| 40 | Dan4589 | 140 | Shanhan8675 | 240 | Taishan1hao |  |
| 41 | Dan6172 | 141 | Shanyou225 | 241 | Xinong1043 |  |
| 42 | Hanxuan1 | 142 | Shi4185 | 242 | Xinmai296 |  |
| 43 | Hanxuan3 | 143 | Shimai12 | 243 | Yunong 416 |  |
| 44 | Hangxuan01 | 144 | Shimai13 | 244 | Yuzhan4 |  |
| 45 | Hangxuan121 | 145 | Shimai18 | 245 | Yunhan2028 |  |
| 46 | Heng136 | 146 | Shimai19 | 246 | Yunhan805 |  |
| 47 | Heng216 | 147 | Shiyou17 | 247 | Chang8744 |  |
| 48 | Heng5229 | 148 | Shiyou20 | 248 | Chang4738 |  |
| 49 | Hengguan35 | 149 | Shunmai1718 | 249 | Changqu521 |  |
| 50 | Hengshui6404 | 150 | Silenghonghulutou | 250 | Zhongmai553 |  |
| 51 | Hengyou18 | 151 | Tai13606 | 251 | Dongxie2 |  |
| 52 | Hongliang4 | 152 | Taiyuan633 | 252 | Hanxuan10hao |  |
| 53 | Huabei187 | 153 | Wanmai 19 | 253 | Hanxuan2hao |  |
| 54 | Huaimai25 | 154 | Weimai4hao | 254 | Heng4399 |  |
| 55 | Huaishu10 | 155 | Wenmai6hao | 255 | Huapei6 |  |
| 56 | Jimai19 | 156 | Xifeng16 | 256 | Jimai21 |  |
| 57 | Jimai229 | 157 | Xifeng20 | 257 | Jimai30 |  |
| 58 | Jimai23 | 158 | Xifeng27 | 258 | Jinmai39 |  |
| 59 | Jimai44 | 159 | Xinong219 | 259 | Jinmai63 |  |
| 60 | Jimai6hao | 160 | Xinong688 | 260 | Luomai9 |  |
| 61 | Jianan17 | 161 | Xiannong4 | 261 | Xindong18 |  |
| 62 | Jimai29 | 162 | Xindong20 | 262 | Yanan15 |  |
| 63 | Jimai2 | 163 | Xindong22 | 263 | 980-4-1-1-2 |  |
| 64 | Jimai32 | 164 | Xuzhou21 | 264 | Boai7023 |  |
| 65 | Jimai38 | 165 | Yannong19 | 265 | Jinan13 |  |
| 66 | Jimai6 | 166 | Yannong21 | 266 | Jimai26 |  |
| 67 | Jimai9 | 167 | Yumai13 | 267 | Jinmai50 |  |
| 68 | Jian26 | 168 | Yumai29 | 268 | Jingshuang16 |  |
| 69 | Jin2148-7 | 169 | Yumai2hao | 269 | Lantian2 |  |
| 70 | Pubing151 | 170 | Yunong949 | 270 | Liangxing99 |  |
| 71 | Jinmai13 | 171 | Yuandong3hao | 271 | Linfeng518 |  |
| 72 | Jinmai16 | 172 | Yunhan102 | 272 | Linfeng615 |  |
| 73 | Jinmai25 | 173 | Yunhan115 | 273 | Linhan51241 |  |
| 74 | Jinmai33 | 174 | Yunhan20410 | 274 | Linhan6hao |  |
| 75 | Jinmai40 | 175 | Yunhan21-30 | 275 | Longjian101 |  |
| 76 | Jinmai44 | 176 | Yunhan22-33 | 276 | Lude1hao |  |
| 77 | Jinmai47 | 177 | Yunhan618 | 277 | Lumai1hao |  |
| 78 | Jinmai51 | 178 | Yunhan719 | 278 | Neimai11 |  |
| 79 | Jinmai53 | 179 | Chang6154 | 279 | Nongda311 |  |
| 80 | Jinmai54 | 180 | Chang6359 | 280 | Qingshan821 |  |
| 81 | Jinmai72 | 181 | Chang6452 | 281 | Taishan23 |  |
| 82 | Jinnong207 | 182 | Chang6878 | 282 | Tao157 |  |
| 83 | Jing411 | 183 | Chang7080 | 283 | Xifrng28 |  |
| 84 | Jingdong17 | 184 | Chang844 | 284 | Xinong1018 |  |
| 85 | Jingdong22 | 185 | Changzhi516 | 285 | Xinong189 |  |
| 86 | Jingdong83 Dong65 | 186 | Changzhi620 | 286 | Xinong318 |  |
| 87 | Jingdong8 | 187 | Zhengfeng9962 | 287 | Xinong979 |  |
| 88 | Kangzhuang974 | 188 | Zhenzhou24 | 288 | Yanzhan1hao |  |
| 89 | Kenong199 | 189 | Zhong7902 | 289 | Yumai18 |  |
| 90 | Keyi26 | 190 | Zhongmai175 | 290 | Chang4640 |  |
| 91 | Keyi29 | 191 | Zhongmai36 | 291 | Changzhi6406 |  |
| 92 | Lantian10 | 192 | Zhongmai9hao | 292 | Zhoumai16 |  |
| 93 | Lantian11 | 193 | Zhongyou9507 |  |  |  |
| 94 | Lantian13 | 194 | Zhoumai18 |  |  |  |
| 95 | Lantian18 | 195 | Zhoumai22 |  |  |  |
| 96 | Lantian21 | 196 | Zhoumai23 |  |  |  |
| 97 | Lantian22 | 197 | 0025-17-1 |  |  |  |
| 98 | Lantian28 | 198 | 00-71 |  |  |  |
| 99 | Lantian30 | 199 | 01-368 |  |  |  |
| 100 | Lantian33 | 200 | 02-7 |  |  |  |

**Table S4. Primer sequences used in this study**

| **No.** | **Primer name** | **Primer Sequence** |
| --- | --- | --- |
| 1 | *TaSINA101-CDS-F* | ATGGATGGTGCTACCTCTAGCAA |
| 2 | *TaSINA101-CDS-R* | TACACCGCGTATTGGTTTCAGA |
| 3 | *TaSINA101-KASP-F1* | GAAGGTCGGAGTCAACGGATTATTTACATGACACTAGTAGAGAGCTCAG |
| 4 | *TaSINA101-KASP-F2* | GAAGGTGACCAAGTTCATGCTATTTACATGACACTAGTAGAGAGCTCAT |
| 5 | *TaSINA101-KASP-R* | AACTATACATAATCAATTTTCCTTTCAA |
| 6 | *TaSINA109-KASP-F1* | GAAGGTCGGAGTCAACGGATTACCAGGCTACTATGCTCCTCAC |
| 7 | *TaSINA109-KASP-F2* | GAAGGTGACCAAGTTCATGCTACCAGGCTACTATGCTCCTCAT |
| 8 | *TaSINA109-KASP-R* | TAGTCGGCTGATGACTGATCTATT |
| 9 | *TaSINA1-qPCR-F* | AATGACAAGTACGGATGCGC |
| 10 | *TaSINA1-qPCR-R* | TTGCAGAACATGTAAGCCCG |
| 11 | *TaSINA27 -qPCR-F* | CATTCTGCCCTCTGTGTTGC |
| 12 | *TaSINA27 -qPCR-R* | CATTCTGCCCTCTGTGTTGC |
| 13 | *TaSINA62-qPCR-F* | GCCTCGGCTGCTTATACTGA |
| 14 | *TaSINA62-qPCR-R* | GTGAACCCTTGGCTTGCATC |
| 15 | *TaSINA93 -qPCR-F* | ACTGTGGTGTGCGTTAGGG |
| 16 | *TaSINA93 -qPCR-R* | TACGGATGCACAGATGGAGC |
| 17 | *TaSINA101 -qPCR-F* | CGAAGCTCATCGTTGTCTGA |
| 18 | *TaSINA101 -qPCR-R* | TACACCGCGTATTGGTTTCA |
| 19 | *TaSINA129 -qPCR-F* | ACTGGACAGCAAGTGCCATT |
| 20 | *TaSINA129 -qPCR-R* | CAGAAGCATGGGGCGTATGA |
| 21 | *TaActin-qPCR-F* | GACCCAGACAACTCGCAAC |
| 22 | *TaActin-qPCR-R* | GGAATCCATGACCACCTAC |

**Table S5. Transcriptome data of *TaSINA* family genes in different organs**

| **No.** | **Gene** | **Roots** | | | **Stem** | | | **Leaf** | | | **Spike** | | | **Grain** | | |
| --- | --- | --- | --- | --- | --- | --- | --- | --- | --- | --- | --- | --- | --- | --- | --- | --- |
|  |  | **Flag leaf stage** | **Three Leaf stage** | **Seedling stage** | **Anthesis** | **Two nodes detectable** | **1 cm spike** | **2 dpa** | **Tillering stage** | **Seedling stage** | **Anthesis** | **Flag leaf stage** | **Two nodes detectable** | **30 DAA** | **14 DAA** | **2 DAA** |
| 1 | TaSINA1 | 0.000 | 0.000 | 0.000 | 0.000 | 0.000 | 0.017 | 0.000 | 0.000 | 0.000 | 0.073 | 0.100 | 0.017 | 0.000 | 0.000 | 0.000 |
| 2 | TaSINA2 | 4.715 | 5.533 | 7.852 | 1.364 | 4.481 | 12.342 | 2.658 | 5.382 | 3.427 | 15.173 | 15.660 | 31.605 | 2.509 | 1.268 | 13.657 |
| 3 | TaSINA3 | 0.000 | 0.000 | 0.000 | 0.018 | 0.000 | 0.000 | 0.000 | 0.000 | 0.000 | 0.052 | 0.092 | 0.000 | 0.000 | 0.044 | 0.058 |
| 4 | TaSINA4 | 0.063 | 0.061 | 0.076 | 0.257 | 0.619 | 0.364 | 0.000 | 0.163 | 0.019 | 6.310 | 2.417 | 2.234 | 0.000 | 0.064 | 3.036 |
| 5 | TaSINA5 | 0.046 | 0.014 | 0.017 | 0.000 | 0.000 | 0.000 | 0.000 | 0.000 | 0.000 | 2.213 | 0.236 | 0.048 | 0.018 | 0.035 | 1.860 |
| 6 | TaSINA6 | 0.076 | 0.066 | 0.057 | 0.000 | 0.000 | 0.000 | 0.000 | 0.000 | 0.000 | 0.015 | 0.000 | 0.007 | 0.174 | 0.016 | 0.008 |
| 7 | TaSINA7 | 0.000 | 0.000 | 0.000 | 0.000 | 0.000 | 0.000 | 0.000 | 0.000 | 0.000 | 0.000 | 0.000 | 0.000 | 0.000 | 0.020 | 0.022 |
| 8 | TaSINA8 | 3.658 | 2.945 | 2.246 | 4.284 | 2.973 | 2.468 | 2.841 | 1.605 | 1.383 | 4.504 | 3.377 | 1.677 | 2.029 | 0.554 | 3.548 |
| 9 | TaSINA9 | 0.000 | 0.006 | 0.000 | 0.334 | 0.060 | 0.037 | 0.000 | 0.036 | 0.123 | 0.052 | 0.020 | 0.000 | 0.000 | 0.000 | 0.000 |
| 10 | TaSINA10 | 2.276 | 2.415 | 1.665 | 0.752 | 2.337 | 2.983 | 0.711 | 1.172 | 0.813 | 4.613 | 4.946 | 7.275 | 1.035 | 0.617 | 4.688 |
| 11 | TaSINA11 | 0.000 | 0.000 | 0.000 | 0.000 | 0.000 | 0.000 | 0.000 | 0.000 | 0.000 | 0.023 | 0.000 | 0.000 | 0.000 | 0.000 | 0.000 |
| 12 | TaSINA12 | 0.635 | 0.623 | 0.635 | 0.014 | 1.767 | 5.057 | 0.076 | 1.257 | 0.628 | 1.749 | 2.134 | 6.800 | 0.396 | 0.342 | 1.227 |
| 13 | TaSINA13 | 0.011 | 0.000 | 0.036 | 0.000 | 0.000 | 0.000 | 0.000 | 0.000 | 0.000 | 0.000 | 0.000 | 0.000 | 0.000 | 0.000 | 0.000 |
| 14 | TaSINA14 | 0.000 | 0.000 | 0.000 | 0.000 | 0.000 | 0.000 | 0.000 | 0.000 | 0.000 | 0.000 | 0.000 | 0.000 | 0.204 | 1.321 | 0.437 |
| 15 | TaSINA15 | 0.022 | 0.016 | 0.000 | 0.000 | 0.000 | 0.044 | 0.068 | 0.036 | 0.000 | 0.272 | 0.088 | 0.032 | 0.169 | 0.000 | 0.000 |
| 16 | TaSINA16 | 0.000 | 0.000 | 0.000 | 0.000 | 0.000 | 0.000 | 0.000 | 0.000 | 0.000 | 0.000 | 0.000 | 0.000 | 0.000 | 0.780 | 0.000 |
| 17 | TaSINA17 | 0.000 | 0.000 | 0.000 | 0.012 | 0.138 | 0.623 | 0.000 | 0.020 | 0.000 | 4.237 | 3.333 | 4.179 | 0.000 | 0.006 | 2.516 |
| 18 | TaSINA18 | 0.039 | 0.042 | 0.009 | 0.131 | 0.066 | 0.516 | 0.000 | 0.250 | 0.065 | 0.829 | 0.559 | 2.036 | 0.000 | 0.010 | 0.826 |
| 19 | TaSINA19 | 0.261 | 0.299 | 0.157 | 0.803 | 1.045 | 2.932 | 0.245 | 1.470 | 0.666 | 7.588 | 3.801 | 7.242 | 1.333 | 0.482 | 4.651 |
| 20 | TaSINA20 | 0.000 | 0.000 | 0.000 | 0.024 | 0.000 | 0.000 | 0.071 | 0.000 | 0.046 | 0.765 | 1.070 | 0.286 | 0.000 | 0.000 | 0.479 |
| 21 | TaSINA21 | 0.000 | 0.000 | 0.000 | 0.000 | 0.000 | 0.000 | 0.000 | 0.000 | 0.000 | 0.022 | 0.067 | 0.000 | 0.000 | 0.000 | 0.024 |
| 22 | TaSINA22 | 5.647 | 4.834 | 5.285 | 4.399 | 4.790 | 7.758 | 3.635 | 3.522 | 2.859 | 54.526 | 10.734 | 10.622 | 5.455 | 0.428 | 4.873 |
| 23 | TaSINA23 | 2.308 | 1.768 | 1.496 | 4.245 | 2.582 | 1.445 | 2.080 | 1.332 | 1.057 | 4.104 | 2.199 | 1.264 | 1.467 | 0.629 | 2.498 |
| 24 | TaSINA24 | 0.000 | 0.000 | 0.000 | 0.036 | 0.174 | 0.095 | 0.000 | 0.046 | 0.056 | 0.306 | 0.098 | 0.369 | 0.010 | 0.070 | 0.140 |
| 25 | TaSINA25 | 0.007 | 0.000 | 0.000 | 0.000 | 0.015 | 0.015 | 0.106 | 0.000 | 0.027 | 0.048 | 0.296 | 0.182 | 0.014 | 0.000 | 0.075 |
| 26 | TaSINA26 | 0.000 | 0.000 | 0.000 | 0.000 | 0.000 | 0.000 | 0.000 | 0.000 | 0.000 | 0.000 | 0.000 | 0.000 | 0.000 | 1.066 | 0.000 |
| 27 | TaSINA27 | 1.269 | 1.206 | 1.009 | 1.840 | 3.159 | 6.096 | 1.650 | 1.972 | 2.382 | 3.041 | 3.426 | 9.243 | 0.588 | 0.445 | 1.457 |
| 28 | TaSINA28 | 0.000 | 0.000 | 0.000 | 0.000 | 0.000 | 0.000 | 0.000 | 0.000 | 0.000 | 0.000 | 0.000 | 0.000 | 0.000 | 1.110 | 0.000 |
| 29 | TaSINA29 | 0.000 | 0.043 | 0.000 | 0.000 | 0.000 | 0.000 | 0.000 | 0.000 | 0.000 | 0.000 | 0.034 | 0.000 | 0.518 | 2.742 | 0.000 |
| 30 | TaSINA30 | 0.070 | 0.110 | 0.075 | 0.000 | 0.056 | 0.351 | 0.000 | 0.000 | 0.000 | 0.000 | 0.424 | 0.446 | 0.018 | 0.008 | 0.019 |
| 31 | TaSINA31 | 0.000 | 0.000 | 0.000 | 0.000 | 0.000 | 0.000 | 0.000 | 0.000 | 0.000 | 0.000 | 0.966 | 0.000 | 0.014 | 0.000 | 0.000 |
| 32 | TaSINA32 | 0.000 | 0.000 | 0.000 | 0.000 | 0.000 | 0.000 | 0.000 | 0.000 | 0.000 | 0.000 | 0.049 | 0.000 | 0.000 | 0.000 | 0.000 |
| 33 | TaSINA33 | 0.000 | 0.000 | 0.000 | 0.000 | 0.000 | 0.000 | 0.000 | 0.000 | 0.000 | 0.140 | 0.000 | 0.000 | 0.000 | 0.000 | 0.000 |
| 34 | TaSINA34 | 0.086 | 0.038 | 0.049 | 0.000 | 0.070 | 0.196 | 0.000 | 0.039 | 0.067 | 1.413 | 1.329 | 2.155 | 0.042 | 0.000 | 0.691 |
| 35 | TaSINA35 | 0.000 | 0.000 | 0.000 | 0.022 | 0.000 | 0.000 | 0.000 | 0.000 | 0.000 | 0.000 | 0.000 | 0.000 | 0.000 | 0.000 | 0.000 |
| 36 | TaSINA36 | 0.000 | 0.000 | 0.000 | 0.000 | 0.000 | 0.000 | 0.000 | 0.000 | 0.000 | 0.000 | 0.051 | 0.000 | 0.000 | 0.000 | 0.000 |
| 37 | TaSINA37 | 0.000 | 0.000 | 0.000 | 0.000 | 0.000 | 0.000 | 0.000 | 0.000 | 0.000 | 0.017 | 0.000 | 0.000 | 0.000 | 0.000 | 0.000 |
| 38 | TaSINA38 | 0.040 | 0.016 | 0.129 | 0.000 | 0.022 | 0.206 | 0.000 | 0.035 | 0.350 | 0.204 | 0.200 | 0.179 | 0.020 | 0.010 | 0.266 |
| 39 | TaSINA39 | 0.000 | 0.000 | 0.011 | 0.000 | 0.000 | 0.000 | 0.000 | 0.013 | 0.026 | 0.878 | 0.884 | 0.170 | 0.000 | 0.007 | 0.708 |
| 40 | TaSINA40 | 0.000 | 0.000 | 0.000 | 0.000 | 0.000 | 0.000 | 0.000 | 0.000 | 0.000 | 0.000 | 1.208 | 0.000 | 0.000 | 0.000 | 0.000 |
| 41 | TaSINA41 | 4.038 | 3.674 | 3.361 | 5.265 | 4.627 | 5.240 | 4.369 | 4.030 | 2.841 | 47.404 | 8.048 | 6.624 | 3.044 | 0.428 | 3.699 |
| 42 | TaSINA42 | 4.644 | 4.765 | 4.943 | 4.423 | 8.267 | 11.553 | 3.190 | 2.820 | 1.980 | 53.404 | 15.498 | 13.817 | 6.541 | 0.830 | 6.823 |
| 43 | TaSINA43 | 0.495 | 0.475 | 0.734 | 0.147 | 0.965 | 3.120 | 0.081 | 0.972 | 1.354 | 1.658 | 2.474 | 6.267 | 0.074 | 0.267 | 0.645 |
| 44 | TaSINA44 | 0.000 | 0.005 | 0.000 | 0.000 | 0.000 | 0.000 | 0.000 | 0.000 | 0.000 | 0.033 | 0.008 | 0.000 | 0.015 | 2.410 | 0.028 |
| 45 | TaSINA45 | 6.963 | 7.078 | 8.540 | 15.984 | 13.271 | 11.311 | 0.832 | 4.544 | 6.357 | 11.122 | 13.110 | 13.205 | 20.187 | 1.114 | 9.075 |
| 46 | TaSINA46 | 0.000 | 0.000 | 0.012 | 0.090 | 0.014 | 0.023 | 0.501 | 0.012 | 0.012 | 3.516 | 3.602 | 2.505 | 0.000 | 0.018 | 4.117 |
| 47 | TaSINA47 | 0.000 | 0.000 | 0.000 | 0.000 | 0.000 | 0.000 | 0.000 | 0.000 | 0.000 | 0.034 | 0.000 | 0.000 | 0.015 | 0.105 | 0.601 |
| 48 | TaSINA48 | 0.000 | 0.030 | 0.019 | 0.000 | 0.000 | 0.000 | 0.000 | 0.000 | 0.000 | 0.000 | 0.000 | 0.000 | 0.020 | 0.000 | 0.000 |
| 49 | TaSINA49 | 0.000 | 0.000 | 0.000 | 0.000 | 0.000 | 0.000 | 0.806 | 0.259 | 0.000 | 0.000 | 0.000 | 0.000 | 0.000 | 0.074 | 0.090 |
| 50 | TaSINA50 | 11.756 | 10.483 | 12.627 | 8.058 | 10.853 | 10.233 | 6.866 | 5.526 | 5.141 | 11.064 | 10.736 | 10.142 | 5.668 | 1.619 | 11.441 |
| 51 | TaSINA51 | 3.440 | 5.072 | 5.098 | 1.927 | 4.431 | 9.765 | 1.539 | 4.617 | 2.515 | 11.648 | 14.547 | 26.584 | 2.601 | 1.185 | 8.962 |
| 52 | TaSINA52 | 0.029 | 0.014 | 0.045 | 0.100 | 0.231 | 0.246 | 0.000 | 0.151 | 0.019 | 1.383 | 0.477 | 1.211 | 0.009 | 0.081 | 0.581 |
| 53 | TaSINA53 | 0.000 | 0.000 | 0.000 | 0.000 | 0.000 | 0.000 | 0.000 | 0.000 | 0.000 | 0.000 | 0.000 | 0.000 | 0.034 | 0.359 | 0.000 |
| 54 | TaSINA54 | 0.000 | 0.000 | 0.000 | 0.046 | 0.091 | 0.061 | 0.000 | 0.029 | 0.027 | 4.374 | 1.923 | 1.714 | 0.013 | 0.008 | 0.836 |
| 55 | TaSINA55 | 2.441 | 1.793 | 2.057 | 1.391 | 1.917 | 2.554 | 2.358 | 1.057 | 0.689 | 3.173 | 3.307 | 3.716 | 5.902 | 0.974 | 3.729 |
| 56 | TaSINA56 | 0.219 | 0.249 | 0.063 | 0.000 | 0.020 | 0.012 | 0.753 | 0.159 | 0.010 | 0.010 | 0.011 | 0.000 | 0.000 | 0.036 | 0.075 |
| 57 | TaSINA57 | 0.157 | 0.191 | 0.232 | 0.084 | 0.581 | 1.168 | 0.039 | 0.235 | 0.136 | 1.628 | 0.706 | 1.020 | 0.082 | 0.051 | 1.664 |
| 58 | TaSINA58 | 0.129 | 0.040 | 0.000 | 0.058 | 0.023 | 0.000 | 0.000 | 0.023 | 0.053 | 0.644 | 0.149 | 0.028 | 0.428 | 0.000 | 1.962 |
| 59 | TaSINA59 | 0.000 | 0.010 | 0.000 | 2.153 | 0.282 | 0.000 | 0.729 | 0.321 | 0.997 | 0.861 | 0.072 | 0.083 | 0.040 | 0.020 | 0.627 |
| 60 | TaSINA60 | 3.367 | 2.727 | 2.789 | 1.202 | 2.234 | 3.747 | 2.777 | 1.511 | 1.071 | 4.735 | 4.022 | 6.013 | 6.668 | 1.214 | 5.082 |
| 61 | TaSINA61 | 0.220 | 0.183 | 0.172 | 0.428 | 0.402 | 0.482 | 0.293 | 0.352 | 0.206 | 0.664 | 0.437 | 0.572 | 0.311 | 0.130 | 0.324 |
| 62 | TaSINA62 | 0.000 | 0.000 | 0.000 | 0.016 | 0.000 | 0.090 | 0.000 | 0.000 | 0.000 | 0.054 | 0.162 | 0.102 | 0.101 | 0.000 | 0.000 |
| 63 | TaSINA63 | 0.037 | 0.021 | 0.033 | 0.019 | 0.056 | 0.174 | 0.000 | 0.018 | 0.018 | 0.106 | 0.077 | 0.304 | 0.000 | 0.009 | 0.174 |
| 64 | TaSINA64 | 0.060 | 0.005 | 0.000 | 0.526 | 0.107 | 0.059 | 0.125 | 0.023 | 0.059 | 13.182 | 5.525 | 2.145 | 0.082 | 0.284 | 7.331 |
| 65 | TaSINA65 | 0.000 | 0.000 | 0.000 | 0.000 | 0.000 | 0.000 | 0.000 | 0.000 | 0.000 | 0.000 | 0.000 | 0.010 | 0.000 | 0.000 | 0.000 |
| 66 | TaSINA66 | 5.084 | 4.071 | 5.677 | 3.633 | 13.365 | 21.956 | 3.488 | 8.738 | 8.482 | 6.344 | 20.602 | 36.487 | 5.136 | 1.520 | 3.025 |
| 67 | TaSINA67 | 0.000 | 0.034 | 0.000 | 0.000 | 0.000 | 0.000 | 0.000 | 0.000 | 0.000 | 0.000 | 0.000 | 0.000 | 0.000 | 0.000 | 0.000 |
| 68 | TaSINA68 | 4.421 | 3.447 | 3.278 | 1.050 | 2.661 | 5.036 | 0.182 | 1.121 | 1.304 | 1.080 | 4.575 | 5.623 | 0.584 | 0.679 | 0.624 |
| 69 | TaSINA69 | 0.024 | 0.064 | 0.000 | 0.000 | 0.017 | 0.000 | 0.000 | 0.000 | 0.000 | 4.421 | 3.232 | 2.445 | 0.000 | 0.068 | 2.301 |
| 70 | TaSINA70 | 0.000 | 0.000 | 0.000 | 0.000 | 0.000 | 0.095 | 0.000 | 0.018 | 0.000 | 0.000 | 0.046 | 0.064 | 0.000 | 0.000 | 0.000 |
| 71 | TaSINA71 | 0.000 | 0.000 | 0.000 | 0.000 | 0.000 | 0.000 | 0.000 | 0.000 | 0.000 | 0.000 | 0.000 | 0.010 | 0.000 | 0.000 | 0.000 |
| 72 | TaSINA72 | 0.716 | 1.586 | 0.479 | 2.035 | 0.994 | 0.598 | 1.202 | 0.868 | 0.762 | 2.506 | 1.403 | 0.746 | 0.702 | 0.178 | 1.356 |
| 73 | TaSINA73 | 3.578 | 5.219 | 7.408 | 1.796 | 5.032 | 11.036 | 1.155 | 4.479 | 2.852 | 9.548 | 13.641 | 31.902 | 1.934 | 1.109 | 8.884 |
| 74 | TaSINA74 | 0.123 | 0.091 | 0.000 | 0.000 | 0.000 | 0.000 | 0.000 | 0.000 | 0.000 | 2.641 | 1.092 | 0.631 | 0.000 | 0.000 | 0.104 |
| 75 | TaSINA75 | 0.000 | 0.000 | 0.000 | 0.000 | 0.000 | 0.000 | 0.000 | 0.000 | 0.000 | 0.037 | 0.000 | 0.000 | 0.000 | 0.000 | 0.000 |
| 76 | TaSINA76 | 0.040 | 0.032 | 0.000 | 0.265 | 0.000 | 0.023 | 0.062 | 0.036 | 0.039 | 0.365 | 0.022 | 0.000 | 0.000 | 0.002 | 0.124 |
| 77 | TaSINA77 | 0.443 | 0.603 | 0.369 | 0.537 | 0.928 | 2.632 | 0.345 | 0.970 | 1.745 | 2.516 | 4.118 | 6.212 | 0.880 | 0.263 | 1.010 |
| 78 | TaSINA78 | 0.000 | 0.000 | 0.000 | 0.000 | 0.000 | 0.000 | 0.000 | 0.000 | 0.116 | 0.000 | 0.051 | 0.049 | 0.000 | 0.000 | 0.000 |
| 79 | TaSINA79 | 0.000 | 0.000 | 0.000 | 0.084 | 0.000 | 0.000 | 0.000 | 0.000 | 0.000 | 0.154 | 0.022 | 0.028 | 0.010 | 1.607 | 0.193 |
| 80 | TaSINA80 | 0.000 | 0.000 | 0.000 | 0.000 | 0.000 | 0.000 | 0.000 | 0.000 | 0.000 | 0.000 | 0.049 | 0.000 | 0.000 | 0.000 | 0.051 |
| 81 | TaSINA81 | 2.264 | 2.256 | 1.339 | 0.834 | 8.011 | 2.863 | 1.318 | 1.717 | 0.775 | 2.112 | 2.829 | 6.763 | 0.993 | 0.943 | 7.348 |
| 82 | TaSINA82 | 0.000 | 0.000 | 0.000 | 0.000 | 0.000 | 0.000 | 0.000 | 0.000 | 0.000 | 0.000 | 0.022 | 0.000 | 0.000 | 0.010 | 0.295 |
| 83 | TaSINA83 | 0.023 | 0.000 | 0.000 | 0.044 | 0.024 | 0.000 | 0.042 | 0.018 | 0.000 | 0.025 | 0.022 | 0.010 | 0.000 | 0.010 | 0.046 |
| 84 | TaSINA84 | 0.822 | 0.851 | 0.596 | 0.839 | 0.762 | 1.603 | 0.511 | 0.866 | 0.549 | 2.305 | 1.429 | 1.982 | 3.805 | 0.399 | 1.132 |
| 85 | TaSINA85 | 3.146 | 2.492 | 1.999 | 0.352 | 1.295 | 3.502 | 0.107 | 0.846 | 0.831 | 0.311 | 2.380 | 2.622 | 5.758 | 0.421 | 0.218 |
| 86 | TaSINA86 | 0.000 | 0.000 | 0.000 | 0.000 | 0.000 | 0.000 | 0.000 | 0.000 | 0.000 | 0.009 | 0.000 | 0.009 | 0.000 | 0.000 | 0.000 |
| 87 | TaSINA87 | 0.026 | 0.068 | 0.000 | 0.380 | 0.113 | 0.238 | 0.102 | 0.105 | 0.049 | 4.130 | 2.896 | 2.361 | 1.053 | 1.023 | 4.589 |
| 88 | TaSINA88 | 0.000 | 0.000 | 0.000 | 0.000 | 0.000 | 0.000 | 0.031 | 0.000 | 0.000 | 0.126 | 0.413 | 0.080 | 0.000 | 0.000 | 0.000 |
| 89 | TaSINA89 | 0.163 | 0.164 | 0.332 | 0.647 | 0.508 | 0.894 | 1.914 | 0.191 | 2.009 | 0.429 | 0.216 | 0.174 | 0.496 | 0.024 | 1.248 |
| 90 | TaSINA90 | 0.019 | 0.055 | 0.000 | 0.013 | 0.000 | 0.014 | 0.031 | 0.023 | 0.034 | 1.373 | 0.654 | 0.298 | 0.000 | 0.025 | 0.968 |
| 91 | TaSINA91 | 0.068 | 0.053 | 0.022 | 0.000 | 0.022 | 0.041 | 0.043 | 0.022 | 0.000 | 0.148 | 0.290 | 0.095 | 0.065 | 0.006 | 0.046 |
| 92 | TaSINA92 | 0.020 | 0.026 | 0.000 | 0.000 | 0.050 | 0.105 | 0.000 | 0.000 | 0.037 | 0.098 | 0.190 | 0.688 | 0.000 | 0.019 | 0.316 |
| 93 | TaSINA93 | 3.115 | 2.807 | 1.847 | 1.189 | 2.088 | 3.966 | 1.390 | 1.529 | 1.476 | 6.076 | 7.011 | 8.413 | 2.362 | 1.291 | 5.865 |
| 94 | TaSINA94 | 3.339 | 3.318 | 3.720 | 0.823 | 10.342 | 2.912 | 2.704 | 2.765 | 0.905 | 2.587 | 3.752 | 8.406 | 0.828 | 0.947 | 8.393 |
| 95 | TaSINA95 | 0.011 | 0.004 | 0.000 | 0.022 | 0.000 | 0.020 | 0.034 | 0.021 | 0.000 | 0.053 | 0.258 | 0.093 | 0.000 | 0.000 | 0.091 |
| 96 | TaSINA96 | 0.062 | 0.023 | 0.040 | 0.000 | 0.000 | 0.014 | 0.000 | 0.000 | 0.000 | 0.045 | 0.082 | 0.663 | 0.000 | 0.000 | 0.000 |
| 97 | TaSINA97 | 0.928 | 0.589 | 0.604 | 0.877 | 1.109 | 1.503 | 0.328 | 0.689 | 0.489 | 2.580 | 1.606 | 2.081 | 2.695 | 0.376 | 1.542 |
| 98 | TaSINA98 | 0.859 | 0.423 | 0.884 | 1.069 | 2.493 | 2.480 | 0.153 | 0.650 | 0.856 | 3.175 | 2.271 | 4.359 | 0.232 | 0.233 | 2.706 |
| 99 | TaSINA99 | 0.162 | 0.229 | 0.130 | 0.781 | 0.599 | 0.366 | 0.549 | 0.532 | 0.346 | 1.021 | 0.521 | 0.886 | 1.034 | 0.063 | 0.588 |
| 100 | TaSINA100 | 2.730 | 2.493 | 1.888 | 1.079 | 1.616 | 2.938 | 0.634 | 0.832 | 0.630 | 0.348 | 1.183 | 2.310 | 2.822 | 0.353 | 0.495 |
| 101 | TaSINA101 | 0.485 | 0.460 | 0.548 | 0.452 | 0.676 | 1.257 | 0.286 | 0.788 | 0.289 | 2.964 | 1.731 | 2.285 | 0.469 | 0.191 | 1.966 |
| 102 | TaSINA102 | 0.000 | 0.000 | 0.000 | 0.000 | 0.000 | 0.000 | 0.000 | 0.000 | 0.000 | 0.009 | 0.000 | 0.009 | 0.000 | 0.000 | 0.000 |
| 103 | TaSINA103 | 0.016 | 0.012 | 0.000 | 0.000 | 0.055 | 0.000 | 0.000 | 0.000 | 0.000 | 0.314 | 0.047 | 0.173 | 0.000 | 0.156 | 0.017 |
| 104 | TaSINA104 | 9.007 | 10.839 | 9.960 | 12.165 | 13.195 | 10.214 | 3.785 | 5.225 | 6.355 | 12.224 | 16.132 | 18.178 | 22.650 | 1.247 | 11.436 |
| 105 | TaSINA105 | 0.610 | 0.399 | 0.512 | 1.096 | 1.099 | 0.902 | 0.668 | 0.869 | 0.428 | 1.385 | 1.128 | 1.497 | 0.838 | 0.123 | 0.549 |
| 106 | TaSINA106 | 0.191 | 0.104 | 0.056 | 0.012 | 0.021 | 0.256 | 0.030 | 0.100 | 0.143 | 0.000 | 0.052 | 0.484 | 0.049 | 0.000 | 0.026 |
| 107 | TaSINA107 | 9.769 | 8.979 | 8.119 | 6.400 | 7.402 | 7.284 | 5.951 | 3.631 | 3.335 | 9.593 | 9.368 | 8.887 | 4.624 | 1.417 | 9.452 |
| 108 | TaSINA108 | 0.302 | 0.299 | 0.429 | 0.250 | 0.998 | 1.422 | 0.000 | 0.446 | 0.188 | 2.953 | 2.684 | 3.955 | 0.075 | 0.145 | 1.851 |
| 109 | TaSINA109 | 0.045 | 0.035 | 0.021 | 0.157 | 0.031 | 0.058 | 0.077 | 0.089 | 0.093 | 0.484 | 0.427 | 0.213 | 0.137 | 0.028 | 0.326 |
| 110 | TaSINA110 | 0.281 | 0.279 | 0.283 | 2.000 | 3.271 | 2.799 | 1.149 | 0.609 | 0.366 | 7.082 | 5.773 | 5.913 | 0.786 | 0.829 | 6.388 |
| 111 | TaSINA111 | 5.409 | 6.516 | 4.849 | 1.467 | 0.504 | 0.791 | 8.990 | 2.790 | 1.934 | 1.439 | 0.585 | 0.591 | 1.236 | 0.093 | 0.761 |
| 112 | TaSINA112 | 8.312 | 7.779 | 8.048 | 6.325 | 9.209 | 6.691 | 5.456 | 3.694 | 3.386 | 7.358 | 9.707 | 6.483 | 2.442 | 1.120 | 7.876 |
| 113 | TaSINA113 | 0.000 | 0.000 | 0.000 | 0.000 | 0.000 | 0.077 | 0.000 | 0.000 | 0.019 | 3.389 | 3.037 | 1.541 | 0.000 | 0.000 | 1.739 |
| 114 | TaSINA114 | 0.499 | 0.450 | 0.375 | 0.519 | 1.028 | 1.311 | 0.065 | 0.499 | 0.311 | 2.562 | 1.487 | 3.474 | 0.133 | 0.078 | 2.060 |
| 115 | TaSINA115 | 0.521 | 0.459 | 0.327 | 0.660 | 0.799 | 0.843 | 0.219 | 0.659 | 0.481 | 1.114 | 0.805 | 1.003 | 0.763 | 0.124 | 0.744 |
| 116 | TaSINA116 | 0.241 | 0.192 | 0.068 | 0.311 | 0.672 | 0.847 | 0.581 | 0.173 | 0.158 | 1.673 | 1.916 | 3.140 | 0.346 | 0.137 | 0.544 |
| 117 | TaSINA117 | 1.818 | 1.476 | 4.186 | 7.995 | 7.006 | 2.193 | 4.488 | 2.157 | 2.415 | 5.122 | 2.077 | 4.601 | 8.126 | 0.543 | 5.192 |
| 118 | TaSINA118 | 0.000 | 0.023 | 0.000 | 0.046 | 0.009 | 0.056 | 0.196 | 0.072 | 0.000 | 0.105 | 0.094 | 0.273 | 0.043 | 0.000 | 0.048 |
| 119 | TaSINA119 | 0.000 | 0.000 | 0.000 | 0.000 | 0.000 | 0.000 | 0.000 | 0.000 | 0.000 | 0.000 | 0.000 | 0.000 | 0.000 | 0.188 | 0.000 |
| 120 | TaSINA120 | 0.000 | 0.000 | 0.020 | 0.000 | 0.047 | 0.010 | 0.000 | 0.000 | 0.022 | 2.595 | 1.894 | 0.960 | 0.000 | 0.011 | 0.769 |
| 121 | TaSINA121 | 2.311 | 2.129 | 1.940 | 1.507 | 1.849 | 3.358 | 1.520 | 1.025 | 0.758 | 3.607 | 3.284 | 4.104 | 8.518 | 0.785 | 3.298 |
| 122 | TaSINA122 | 0.000 | 0.000 | 0.000 | 0.000 | 0.000 | 0.026 | 0.000 | 0.000 | 0.000 | 1.412 | 0.921 | 0.564 | 0.000 | 0.000 | 1.688 |
| 123 | TaSINA123 | 2.759 | 2.888 | 2.543 | 0.847 | 10.810 | 3.969 | 1.794 | 2.766 | 1.368 | 2.945 | 3.583 | 9.868 | 1.395 | 0.949 | 11.860 |
| 124 | TaSINA124 | 10.663 | 11.533 | 12.121 | 14.129 | 16.408 | 10.018 | 13.966 | 7.666 | 7.392 | 14.277 | 15.724 | 12.317 | 26.093 | 2.182 | 15.086 |
| 125 | TaSINA125 | 9.322 | 10.086 | 11.720 | 18.474 | 16.737 | 12.599 | 1.598 | 5.339 | 8.260 | 16.047 | 17.884 | 15.928 | 17.297 | 1.414 | 13.404 |
| 126 | TaSINA126 | 5.156 | 5.703 | 4.855 | 7.869 | 8.814 | 6.472 | 7.442 | 4.907 | 4.747 | 9.115 | 9.555 | 8.913 | 11.279 | 1.342 | 8.634 |
| 127 | TaSINA127 | 0.000 | 0.000 | 0.000 | 0.000 | 0.000 | 0.000 | 0.023 | 0.000 | 0.000 | 0.021 | 0.000 | 0.000 | 0.000 | 0.000 | 0.000 |
| 128 | TaSINA128 | 0.000 | 0.000 | 0.000 | 0.083 | 0.013 | 0.000 | 0.000 | 0.019 | 0.023 | 2.803 | 0.923 | 0.709 | 0.000 | 0.000 | 0.593 |
| 129 | TaSINA129 | 1.069 | 0.766 | 1.014 | 0.912 | 1.030 | 1.546 | 0.385 | 0.617 | 0.626 | 2.306 | 1.495 | 1.554 | 3.409 | 0.753 | 1.372 |
| 130 | TaSINA130 | 0.036 | 0.000 | 0.015 | 0.000 | 0.000 | 0.000 | 0.000 | 0.000 | 0.017 | 0.000 | 0.000 | 0.000 | 0.019 | 0.000 | 0.111 |
| 131 | TaSINA131 | 0.000 | 0.045 | 0.013 | 0.000 | 0.056 | 0.288 | 0.000 | 0.022 | 0.027 | 0.051 | 0.126 | 0.730 | 0.000 | 0.000 | 0.424 |

**Table S6. Characteristics of TaSINA family members**

| **Gene name** | **ID** | **Chromosome location** | **Length of coding region (bp)** | **Molecular weight of protein (kD)** |
| --- | --- | --- | --- | --- |
| TaSINA1 | TraesCS1A02G060800 | 1A:41758210-41759420 | 924 | 33.62 |
| TaSINA2 | TraesCS2D02G131700 | 2D:76761118-76763951 | 906 | 34.18 |
| TaSINA3 | TraesCS3D02G029700 | 3D:10723713-10724965 | 858 | 29.73 |
| TaSINA4 | TraesCS7B02G040200 | 7B:40286001-40287760 | 951 | 33.04 |
| TaSINA5 | TraesCS7A02G138000 | 7A:90478930-90481541 | 951 | 33.03 |
| TaSINA6 | TraesCS7D02G071600 | 7D:42184172-42195293 | 900 | 32.04 |
| TaSINA7 | TraesCS3B02G033800 | 3B:16133129-16137429 | 825 | 30.59 |
| TaSINA8 | TraesCS4B02G363200 | 4B:652001040-652003347 | 987 | 35.60 |
| TaSINA9 | TraesCS3B02G028000 | 3B:12108159-12109513 | 795 | 27.79 |
| TaSINA10 | TraesCS6D02G324500 | 6D:430946344-430948047 | 714 | 37.26 |
| TaSINA11 | TraesCS3B02G052700 | 3B:26683993-26684842 | 762 | 28.29 |
| TaSINA12 | TraesCS3B02G028900 | 3B:12404016-12408859 | 906 | 32.55 |
| TaSINA13 | TraesCS3B02G560800 | 3B:794632929-794634205 | 837 | 30.97 |
| TaSINA14 | TraesCS3A02G048300 | 3A:25871914-25876451 | 825 | 30.65 |
| TaSINA15 | TraesCS4D02G110300 | 4D:90926162-90927074 | 831 | 30.29 |
| TaSINA16 | TraesCS3B02G030800 | 3B:14022297-14023737 | 882 | 30.83 |
| TaSINA17 | TraesCSU02G043700 | Un:35207902-35209689 | 951 | 33.68 |
| TaSINA18 | TraesCS3D02G029300 | 3D:10642509-10644759 | 1023 | 37.86 |
| TaSINA19 | TraesCS3A02G032200 | 3A:17621292-17623525 | 1023 | 38.08 |
| TaSINA20 | TraesCS3D02G017100 | 3D:5861814-5863543 | 957 | 33.32 |
| TaSINA21 | TraesCS3B02G074800 | 3B:45541948-45543229 | 834 | 30.73 |
| TaSINA22 | TraesCS3D02G165600 | 3D:137485298-137488207 | 978 | 36.17 |
| TaSINA23 | TraesCS4D02G356400 | 4D:505352502-505354843 | 990 | 35.60 |
| TaSINA24 | TraesCS3B02G053500 | 3B:27376747-27379985 | 849 | 31.11 |
| TaSINA25 | TraesCSU02G050700 | Un:40588651-40589980 | 1074 | 37.98 |
| TaSINA26 | TraesCS3B02G054800 | 3B:28408330-28409690 | 984 | 34.64 |
| TaSINA27 | TraesCS3B02G054400 | 3B:28102448-28105701 | 894 | 32.25 |
| TaSINA28 | TraesCS3B02G055000 | 3B:28454847-28456101 | 984 | 34.73 |
| TaSINA29 | TraesCS5D02G504400 | 5D:530841120-530841725 | 606 | 22.01 |
| TaSINA30 | TraesCSU02G178600 | Un:269094353-269095487 | 927 | 33.24 |
| TaSINA31 | TraesCSU02G231400 | Un:342603523-342604885 | 1041 | 39.66 |
| TaSINA32 | TraesCS3D02G030300 | 3D:11206846-11208625 | 900 | 32.90 |
| TaSINA33 | TraesCSU02G046100 | Un:36295496-36296690 | 948 | 34.24 |
| TaSINA34 | TraesCS3B02G079000 | 3B:49866399-49867785 | 936 | 32.23 |
| TaSINA35 | TraesCS3A02G021100 | 3A:12173882-12175034 | 828 | 24.10 |
| TaSINA36 | TraesCS3A02G028900 | 3A:15314855-15315997 | 1062 | 37.35 |
| TaSINA37 | TraesCS3B02G027500 | 3B:11927748-11928939 | 945 | 34.04 |
| TaSINA38 | TraesCS3B02G033700 | 3B:16129137-16130949 | 879 | 32.34 |
| TaSINA39 | TraesCS7D02G129300 | 7D:81759599-81760943 | 969 | 34.17 |
| TaSINA40 | TraesCS5A02G228300 | 5A:444548210-444549501 | 947 | 34.51 |
| TaSINA41 | TraesCS3B02G185000 | 3B:200873039-200875826 | 984 | 36.28 |
| TaSINA42 | TraesCS3A02G158000 | 3A:155429154-155431604 | 939 | 34.74 |
| TaSINA43 | TraesCS3A02G030100 | 3A:15783983-15788659 | 894 | 31.95 |
| TaSINA44 | TraesCS3D02G028400 | 3D:9947161-9949118 | 975 | 34.00 |
| TaSINA45 | TraesCSU02G019700 | Un:21199465-21205177 | 936 | 34.83 |
| TaSINA46 | TraesCS3A02G028800 | 3A:15302528-15304140 | 963 | 33.43 |
| TaSINA47 | TraesCS3D02G031300 | 3D:11445394-11449904 | 825 | 30.58 |
| TaSINA48 | TraesCS2A02G446400 | 2A:695505011-695505933 | 810 | 29.37 |
| TaSINA49 | TraesCS3A02G101600 | 3A:65995530-65996144 | 534 | 19.78 |
| TaSINA50 | TraesCS7D02G340900 | 7D:436736334-436740756 | 1026 | 37.67 |
| TaSINA51 | TraesCS2B02G151100 | 2B:118060595-118063455 | 1053 | 39.46 |
| TaSINA52 | TraesCS3B02G052400 | 3B:26586965-26589238 | 1023 | 37.89 |
| TaSINA53 | TraesCS3B02G054900 | 3B:28421665-28422753 | 984 | 34.69 |
| TaSINA54 | TraesCS3B02G079100 | 3B:50020798-50022010 | 978 | 33.56 |
| TaSINA55 | TraesCS2D02G600200 | 2D:650962449-650964288 | 1098 | 41.32 |
| TaSINA56 | TraesCS3A02G101400 | 3A:65952061-65953597 | 630 | 23.54 |
| TaSINA57 | TraesCS3A02G062800 | 3A:37507075-37508070 | 873 | 31.92 |
| TaSINA58 | TraesCS7B02G147600 | 7B:193949873-193950559 | 687 | 24.41 |
| TaSINA59 | TraesCS3A02G027900 | 3A:14857357-14859427 | 819 | 29.58 |
| TaSINA60 | TraesCS2A02G586400 | 2A:777064713-777066560 | 1098 | 41.39 |
| TaSINA61 | TraesCS3A02G030600 | 3A:16620265-16622563 | 849 | 31.25 |
| TaSINA62 | TraesCS4A02G148400 | 4A:279467861-279470964 | 771 | 29.00 |
| TaSINA63 | TraesCS3B02G019600 | 3B:8490844-8495554 | 951 | 34.34 |
| TaSINA64 | TraesCS3A02G030000 | 3A:15779481-15780912 | 996 | 33.93 |
| TaSINA65 | TraesCS3A02G020900 | 3A:12167514-12168498 | 897 | 32.05 |
| TaSINA66 | TraesCSU02G045900 | Un:35884383-35888113 | 900 | 32.35 |
| TaSINA67 | TraesCS3D02G029600 | 3D:10675721-10677063 | 1008 | 37.43 |
| TaSINA68 | TraesCS3D02G512700 | 3D:597095910-597097486 | 1080 | 36.93 |
| TaSINA69 | TraesCS7D02G440300 | 7D:560285786-560287021 | 951 | 33.03 |
| TaSINA70 | TraesCS3D02G504900 | 3D:592523632-592525074 | 825 | 30.22 |
| TaSINA71 | TraesCSU02G040400 | Un:32306421-32307405 | 897 | 32.05 |
| TaSINA72 | TraesCS5A02G531400 | 5A:689617846-689620273 | 759 | 28.16 |
| TaSINA73 | TraesCS2A02G129000 | 2A:77263376-77266187 | 960 | 35.85 |
| TaSINA74 | TraesCS3A02G065700 | 3A:38620950-38622832 | 996 | 34.06 |
| TaSINA75 | TraesCS3D02G016800 | 3D:5812552-5813779 | 945 | 33.80 |
| TaSINA76 | TraesCS3B02G018000 | 3B:7382432-7384235 | 813 | 29.44 |
| TaSINA77 | TraesCS3D02G015600 | 3D:5601666-5605871 | 894 | 32.15 |
| TaSINA78 | TraesCS3B02G050200 | 3B:25179852-25181748 | 744 | 27.16 |
| TaSINA79 | TraesCS3D02G028300 | 3D:9880635-9882400 | 981 | 34.08 |
| TaSINA80 | TraesCS3A02G031100 | 3A:16927637-16929900 | 810 | 30.35 |
| TaSINA81 | TraesCS2A02G589200 | 2A:778019850-778021071 | 1122 | 41.99 |
| TaSINA82 | TraesCS3B02G031200 | 3B:14201533-14204974 | 879 | 32.23 |
| TaSINA83 | TraesCS4B02G109100 | 4B:121451470-121452382 | 831 | 30.36 |
| TaSINA84 | TraesCS3A02G032100 | 3A:17604492-17607584 | 1371 | 51.00 |
| TaSINA85 | TraesCS3A02G505400 | 3A:727224168-727225756 | 1080 | 36.95 |
| TaSINA86 | TraesCSU02G040300 | Un:32299007-32299999 | 669 | 24.10 |
| TaSINA87 | TraesCS3D02G018300 | 3D:6167260-6169993 | 972 | 35.03 |
| TaSINA88 | TraesCS3D02G017000 | 3D:5856669-5858004 | 1098 | 38.66 |
| TaSINA89 | TraesCS1D02G102900 | 1D:92234960-92236394 | 1149 | 40.49 |
| TaSINA90 | TraesCS7B02G030300 | 7B:28686332-28687723 | 969 | 34.33 |
| TaSINA91 | TraesCS3B02G027000 | 3B:11663786-11665319 | 1074 | 37.94 |
| TaSINA92 | TraesCS3B02G050900 | 3B:25416412-25417913 | 879 | 31.78 |
| TaSINA93 | TraesCS6A02G343600 | 6A:577780417-577781984 | 1044 | 37.25 |
| TaSINA94 | TraesCS2D02G564800 | 2D:635092283-635094004 | 1128 | 42.07 |
| TaSINA95 | TraesCS1D02G061700 | 1D:41756175-41757926 | 1059 | 39.35 |
| TaSINA96 | TraesCS3D02G066100 | 3D:29281632-29282828 | 942 | 32.44 |
| TaSINA97 | TraesCS3D02G029400 | 3D:10659758-10663012 | 1380 | 51.34 |
| TaSINA98 | TraesCS3A02G025200 | 3A:13404835-13407376 | 825 | 29.81 |
| TaSINA99 | TraesCS3B02G053000 | 3B:26757094-26759582 | 846 | 31.40 |
| TaSINA100 | TraesCS3B02G572900 | 3B:803628595-803630088 | 1089 | 37.08 |
| TaSINA101 | TraesCS3B02G052800 | 3B:26746166-26748742 | 849 | 31.44 |
| TaSINA102 | TraesCSU02G177200 | Un:267482452-267483444 | 669 | 24.10 |
| TaSINA103 | TraesCS4A02G395500 | 4A:670215256-670216736 | 840 | 31.00 |
| TaSINA104 | TraesCS6A02G086800 | 6A:55545195-55550804 | 936 | 34.86 |
| TaSINA105 | TraesCS3D02G029000 | 3D:10458906-10461617 | 849 | 31.31 |
| TaSINA106 | TraesCS4D02G164700 | 4D:266783514-266785811 | 906 | 33.87 |
| TaSINA107 | TraesCS7B02G244800 | 7B:453149024-453153355 | 1026 | 37.62 |
| TaSINA108 | TraesCS3B02G031100 | 3B:14162064-14164236 | 1218 | 44.48 |
| TaSINA109 | TraesCS1A02G070000 | 1A:52540443-52542008 | 732 | 25.83 |
| TaSINA110 | TraesCS3A02G027500 | 3A:14764482-14766910 | 831 | 29.63 |
| TaSINA111 | TraesCS1A02G371400 | 1A:548470147-548471997 | 603 | 22.57 |
| TaSINA112 | TraesCS7A02G332700 | 7A:486142143-486146449 | 1026 | 37.67 |
| TaSINA113 | TraesCS7B02G351200 | 7B:608300510-608301600 | 927 | 32.30 |
| TaSINA114 | TraesCS3D02G020800 | 3D:6974954-6977481 | 831 | 30.20 |
| TaSINA115 | TraesCS3D02G028800 | 3D:10409012-10411521 | 645 | 23.74 |
| TaSINA116 | TraesCS3B02G442300 | 3B:681568413-681570632 | 825 | 29.54 |
| TaSINA117 | TraesCS1A02G136400 | 1A:216414924-216416041 | 678 | 25.76 |
| TaSINA118 | TraesCS3B02G050700 | 3B:25283156-25285606 | 924 | 33.63 |
| TaSINA119 | TraesCS3B02G055100 | 3B:28517512-28518914 | 984 | 34.85 |
| TaSINA120 | TraesCS7A02G451200 | 7A:644526016-644527579 | 954 | 33.21 |
| TaSINA121 | TraesCSU02G021700 | Un:23530582-23532441 | 1098 | 41.37 |
| TaSINA122 | TraesCS3B02G026900 | 3B:11659067-11660490 | 963 | 33.42 |
| TaSINA123 | TraesCS2B02G594500 | 2B:779101022-779102772 | 1128 | 42.15 |
| TaSINA124 | TraesCS1D02G134000 | 1D:165203036-165203912 | 759 | 28.90 |
| TaSINA125 | TraesCS6B02G114800 | 6B:98851011-98857015 | 936 | 34.83 |
| TaSINA126 | TraesCS1B02G152800 | 1B:246768104-246800383 | 1224 | 45.16 |
| TaSINA127 | TraesCS3D02G018100 | 3D:6140574-6142644 | 807 | 29.13 |
| TaSINA128 | TraesCS3D02G066200 | 3D:29307961-29309437 | 984 | 33.77 |
| TaSINA129 | TraesCS3B02G052300 | 3B:26530785-26532399 | 1395 | 52.04 |
| TaSINA130 | TraesCS3D02G100700 | 3D:53389445-53390512 | 975 | 34.36 |
| TaSINA131 | TraesCS3B02G053400 | 3B:27266173-27269409 | 882 | 32.27 |

**Table S7. Number of haplotypes of *TaSINA* family members**

| **Subfamily** | **Gene Name** | **ID** | **Number of haplotype** | **chromosome** |
| --- | --- | --- | --- | --- |
| I | TaSINA31 | TraesCSUn2G231400 | 0 | Un |
| I | TaSINA55 | TraesCS2D02G600200 | 0 | 2D |
| I | TaSINA60 | TraesCS2A02G586400 | 0 | 2A |
| I | TaSINA81 | TraesCS2A02G589200 | 2 | 2A |
| I | TaSINA94 | TraesCS2D02G564800 | 0 | 2D |
| I | TaSINA121 | TraesCSUn2G021700 | 0 | Un |
| I | TaSINA123 | TraesCS2B02G594500 | 0 | 2B |
| II | TaSINA1 | TraesCS1A02G060800 | 2 | 1A |
| II | TaSINA3 | TraesCS3D02G029700 | 0 | 3D |
| II | TaSINA7 | TraesCS3B02G033800 | 3 | 3B |
| II | TaSINA11 | TraesCS3B02G052700 | 0 | 3B |
| II | TaSINA13 | TraesCS3B02G560800 | 0 | 3B |
| II | TaSINA14 | TraesCS3A02G048300 | 0 | 3A |
| II | TaSINA18 | TraesCS3D02G029300 | 0 | 3D |
| II | TaSINA19 | TraesCS3A02G032200 | 2 | 3A |
| II | TaSINA21 | TraesCS3B02G074800 | 2 | 3B |
| II | TaSINA24 | TraesCS3B02G053500 | 2 | 3B |
| II | TaSINA38 | TraesCS3B02G033700 | 2 | 3B |
| II | TaSINA40 | TraesCS5A02G228300 | 0 | 5A |
| II | TaSINA47 | TraesCS3D02G031300 | 0 | 3D |
| II | TaSINA48 | TraesCS2A02G446400 | 3 | 2A |
| II | TaSINA52 | TraesCS3B02G052400 | 0 | 3B |
| II | TaSINA57 | TraesCS3A02G062800 | 2 | 3A |
| II | TaSINA61 | TraesCS3A02G030600 | 2 | 3A |
| II | TaSINA67 | TraesCS3D02G029600 | 0 | 3D |
| II | TaSINA70 | TraesCS3D02G504900 | 0 | 3D |
| II | TaSINA78 | TraesCS3B02G050200 | 2 | 3B |
| II | TaSINA80 | TraesCS3A02G031100 | 2 | 3A |
| II | TaSINA82 | TraesCS3B02G031200 | 2 | 3B |
| II | TaSINA84 | TraesCS3A02G032100 | 2 | 3A |
| II | TaSINA92 | TraesCS3B02G050900 | 2 | 3B |
| II | TaSINA95 | TraesCS1D02G061700 | 2 | 1D |
| II | TaSINA97 | TraesCS3D02G029400 | 0 | 3D |
| II | TaSINA99 | TraesCS3B02G053000 | 2 | 3B |
| II | TaSINA101 | TraesCS3B02G052800 | 2 | 3B |
| II | TaSINA105 | TraesCS3D02G029000 | 0 | 3D |
| II | TaSINA108 | TraesCS3B02G031100 | 3 | 3B |
| II | TaSINA109 | TraesCS1A02G070000 | 2 | 1A |
| II | TaSINA118 | TraesCS3B02G050700 | 0 | 3B |
| II | TaSINA129 | TraesCS3B02G052300 | 0 | 3B |
| II | TaSINA131 | TraesCS3B02G053400 | 3 | 3B |
| II | TaSINA32 | TraesCS3D02G030300 | 2 | 3D |
| II | TaSINA89 | TraesCS1D02G102900 | 0 | 1D |
| II | TaSINA115 | TraesCS3D02G028800 | 0 | 3D |
| III | TaSINA4 | TraesCS7B02G040200 | 2 | 7B |
| III | TaSINA5 | TraesCS7A02G138000 | 0 | 7A |
| III | TaSINA6 | TraesCS7D02G071600 | 0 | 7D |
| III | TaSINA9 | TraesCS3B02G028000 | 3 | 3B |
| III | TaSINA10 | TraesCS6D02G324500 | 0 | 6D |
| III | TaSINA16 | TraesCS3B02G030800 | 2 | 3B |
| III | TaSINA17 | TraesCSUn2G043700 | 0 | Un |
| III | TaSINA20 | TraesCS3D02G017100 | 0 | 3D |
| III | TaSINA25 | TraesCSUn2G050700 | 0 | Un |
| III | TaSINA26 | TraesCS3B02G054800 | 0 | 3B |
| III | TaSINA28 | TraesCS3B02G055000 | 2 | 3B |
| III | TaSINA33 | TraesCSUn2G046100 | 0 | Un |
| III | TaSINA34 | TraesCS3B02G079000 | 0 | 3B |
| III | TaSINA35 | TraesCS3A02G021100 | 2 | 3A |
| III | TaSINA36 | TraesCS3A02G028900 | 2 | 3A |
| III | TaSINA37 | TraesCS3B02G027500 | 2 | 3B |
| III | TaSINA39 | TraesCS7D02G129300 | 0 | 7D |
| III | TaSINA44 | TraesCS3D02G028400 | 0 | 3D |
| III | TaSINA46 | TraesCS3A02G028800 | 2 | 3A |
| III | TaSINA53 | TraesCS3B02G054900 | 0 | 3B |
| III | TaSINA54 | TraesCS3B02G079100 | 2 | 3B |
| III | TaSINA58 | TraesCS7B02G147600 | 0 | 7B |
| III | TaSINA64 | TraesCS3A02G030000 | 3 | 3A |
| III | TaSINA65 | TraesCS3A02G020900 | 3 | 3A |
| III | TaSINA68 | TraesCS3D02G512700 | 2 | 3D |
| III | TaSINA69 | TraesCS7D02G440300 | 2 | 7D |
| III | TaSINA71 | TraesCSUn2G040400 | 0 | Un |
| III | TaSINA74 | TraesCS3A02G065700 | 0 | 3A |
| III | TaSINA75 | TraesCS3D02G016800 | 0 | 3D |
| III | TaSINA79 | TraesCS3D02G028300 | 0 | 3D |
| III | TaSINA83 | TraesCS4B02G109100 | 0 | 4B |
| III | TaSINA85 | TraesCS3A02G505400 | 2 | 3A |
| III | TaSINA86 | TraesCSUn2G040300 | 0 | Un |
| III | TaSINA88 | TraesCS3D02G017000 | 0 | 3D |
| III | TaSINA90 | TraesCS7B02G030300 | 0 | 7B |
| III | TaSINA91 | TraesCS3B02G027000 | 0 | 3B |
| III | TaSINA93 | TraesCS6A02G343600 | 2 | 6A |
| III | TaSINA96 | TraesCS3D02G066100 | 0 | 3D |
| III | TaSINA100 | TraesCS3B02G572900 | 2 | 3B |
| III | TaSINA102 | TraesCSUn2G177200 | 0 | Un |
| III | TaSINA113 | TraesCS7B02G351200 | 0 | 7B |
| III | TaSINA119 | TraesCS3B02G055100 | 0 | 3B |
| III | TaSINA120 | TraesCS7A02G451200 | 3 | 7A |
| III | TaSINA122 | TraesCS3B02G026900 | 3 | 3B |
| III | TaSINA128 | TraesCS3D02G066200 | 2 | 3D |
| III | TaSINA130 | TraesCS3D02G100700 | 0 | 3D |
| III | TaSINA15 | TraesCS4D02G110300 | 0 | 4D |
| III | TaSINA30 | TraesCSUn2G178600 | 0 | Un |
| IV | TaSINA12 | TraesCS3B02G028900 | 2 | 3B |
| IV | TaSINA27 | TraesCS3B02G054400 | 0 | 3B |
| IV | TaSINA43 | TraesCS3A02G030100 | 4 | 3A |
| IV | TaSINA66 | TraesCSUn2G045900 | 0 | Un |
| IV | TaSINA77 | TraesCS3D02G015600 | 2 | 3D |
| V | TaSINA8 | TraesCS4B02G363200 | 0 | 4B |
| V | TaSINA23 | TraesCS4D02G356400 | 0 | 4D |
| V | TaSINA29 | TraesCS5D02G504400 | 0 | 5D |
| V | TaSINA49 | TraesCS3A02G101600 | 3 | 3A |
| V | TaSINA56 | TraesCS3A02G101400 | 2 | 3A |
| V | TaSINA59 | TraesCS3A02G027900 | 0 | 3A |
| V | TaSINA63 | TraesCS3B02G019600 | 2 | 3B |
| V | TaSINA72 | TraesCS5A02G531400 | 2 | 5A |
| V | TaSINA76 | TraesCS3B02G018000 | 2 | 3B |
| V | TaSINA87 | TraesCS3D02G018300 | 0 | 3D |
| V | TaSINA98 | TraesCS3A02G025200 | 2 | 3A |
| V | TaSINA103 | TraesCS4A02G395500 | 0 | 4A |
| V | TaSINA110 | TraesCS3A02G027500 | 2 | 3A |
| V | TaSINA114 | TraesCS3D02G020800 | 0 | 3D |
| V | TaSINA116 | TraesCS3B02G442300 | 0 | 3B |
| V | TaSINA127 | TraesCS3D02G018100 | 0 | 3D |
| VI | TaSINA2 | TraesCS2D02G131700 | 2 | 2D |
| VI | TaSINA22 | TraesCS3D02G165600 | 2 | 3D |
| VI | TaSINA41 | TraesCS3B02G185000 | 2 | 3B |
| VI | TaSINA42 | TraesCS3A02G158000 | 0 | 3A |
| VI | TaSINA45 | TraesCSUn2G019700 | 0 | Un |
| VI | TaSINA50 | TraesCS7D02G340900 | 0 | 7D |
| VI | TaSINA62 | TraesCS4A02G148400 | 2 | 4A |
| VI | TaSINA73 | TraesCS2A02G129000 | 2 | 2A |
| VI | TaSINA104 | TraesCS6A02G086800 | 0 | 6A |
| VI | TaSINA106 | TraesCS4D02G164700 | 0 | 4D |
| VI | TaSINA107 | TraesCS7B02G244800 | 2 | 7B |
| VI | TaSINA111 | TraesCS1A02G371400 | 2 | 1A |
| VI | TaSINA112 | TraesCS7A02G332700 | 3 | 7A |
| VI | TaSINA117 | TraesCS1A02G136400 | 0 | 1A |
| VI | TaSINA124 | TraesCS1D02G134000 | 0 | 1D |
| VI | TaSINA125 | TraesCS6B02G114800 | 2 | 6B |
| VI | TaSINA126 | TraesCS1B02G152800 | 3 | 1B |
| VI | TaSINA51 | TraesCS2B02G151100 | 0 | 2B |

**Table S8. Association analysis of TGW, GL and GW with different haplotypes of five *TaSINA* genes**

| **Gene name** | **Years and locations** | **Grain traits** | **Haplotypes** | **Mean±SE** | **Significance** |
| --- | --- | --- | --- | --- | --- |
| *TaSINA56* | 2002LY | TGW | *HapⅠ* | 34.186±0.896 | *** |
|  |  |  | *HapⅡ* | 43.13±0.948 |  |
|  | 2005LY | TGW | *HapⅠ* | 31.935±0.927 | *** |
|  |  |  | *HapⅡ* | 39.868±0.84 |  |
|  | 2010SY | TGW | *HapⅠ* | 32.799±0.817 | *** |
|  |  |  | *HapⅡ* | 40.936±0.81 |  |
|  | 2002LY | GL | *HapⅠ* | 0.617±0.008 | *** |
|  |  |  | *HapⅡ* | 0.675±0.008 |  |
|  | 2005LY | GL | *HapⅠ* | 0.627±0.007 | *** |
|  |  |  | *HapⅡ* | 0.684±0.007 |  |
|  | 2010SY | GL | *HapⅠ* | 0.684±0.007 | *** |
|  |  |  | *HapⅡ* | 0.653±0.007 |  |
|  | 2002LY | GW | *HapⅠ* | 0.302±0.005 | *** |
|  |  |  | *HapⅡ* | 0.330±0.003 |  |
|  | 2005LY | GW | *HapⅠ* | 0.298±0.003 | *** |
|  |  |  | *HapⅡ* | 0.325±0.003 |  |
|  | 2010SY | GW | *HapⅠ* | 0.301±0.003 | *** |
|  |  |  | *HapⅡ* | 0.327±0.003 |  |
| *TaSINA62* | 2002LY | TGW | *HapⅠ* | 39.398±0.809 | * |
|  |  |  | *HapⅡ* | 33.963±2.182 |  |
|  | 2005LY | TGW | *HapⅠ* | 36.805±0.782 | ** |
|  |  |  | *HapⅡ* | 30.035±1.837 |  |
|  | 2010SY | TGW | *HapⅠ* | 37.243±0.739 | ns |
|  |  |  | *HapⅡ* | 33.234±2.194 |  |
|  | 2002LY | GL | *HapⅠ* | 0.641±0.007 | ns |
|  |  |  | *HapⅡ* | 0.64±0.014 |  |
|  | 2005LY | GL | *HapⅠ* | 0.656±0.006 | ns |
|  |  |  | *HapⅡ* | 0.643±0.02 |  |
|  | 2010SY | GL | *HapⅠ* | 0.675±0.006 | ns |
|  |  |  | *HapⅡ* | 2.666±0.015 |  |
|  | 2002LY | GW | *HapⅠ* | 0.321±0.003 | * |
|  |  |  | *HapⅡ* | 0.302±0.007 |  |
|  | 2005LY | GW | *HapⅠ* | 0.316±0.002 | *** |
|  |  |  | *HapⅡ* | 0.289±0.007 |  |
|  | 2010SY | GW | *HapⅠ* | 0.316±0.002 | ** |
|  |  |  | *HapⅡ* | 0.297±0.006 |  |
| *TaSINA101* | 2002LY | TGW | *HapⅠ* | 37.102±0.861 | ** |
|  |  |  | *HapⅡ* | 43.474±2.204 |  |
|  | 2005LY | TGW | *HapⅠ* | 35.087±0.82 | * |
|  |  |  | *HapⅡ* | 40.206±1.996 |  |
|  | 2010SY | TGW | *HapⅠ* | 35.86±0.762 | * |
|  |  |  | *HapⅡ* | 40.969±1.876 |  |
|  | 2002LY | GL | *HapⅠ* | 0.641±0.007 | ns |
|  |  |  | *HapⅡ* | 0.652±0.013 |  |
|  | 2005LY | GL | *HapⅠ* | 0.651±0.007 | ns |
|  |  |  | *HapⅡ* | 0.679±0.014 |  |
|  | 2010SY | GL | *HapⅠ* | 0.673±0.006 | ns |
|  |  |  | *HapⅡ* | 0.682±0.012 |  |
|  | 2002LY | GW | *HapⅠ* | 0.312±0.004 | ns |
|  |  |  | *HapⅡ* | 0.332±0.009 |  |
|  | 2005LY | GW | *HapⅠ* | 0.309±0.003 | * |
|  |  |  | *HapⅡ* | 0.325±0.005 |  |
|  | 2010SY | GW | *HapⅠ* | 0.311±0.002 | * |
|  |  |  | *HapⅡ* | 0.328±0.006 |  |
| *TaSINA109* | 2002LY | TGW | *HapⅠ* | 35.629±0.872 | *** |
|  |  |  | *HapⅡ* | 43.669±1.024 |  |
|  | 2005LY | TGW | *HapⅠ* | 33.308±0.866 | *** |
|  |  |  | *HapⅡ* | 40.314±0.963 |  |
|  | 2010SY | TGW | *HapⅠ* | 33.896±0.760 | *** |
|  |  |  | *HapⅡ* | 41.111±0.994 |  |
|  | 2002LY | GL | *HapⅠ* | 0.622±0.007 | *** |
|  |  |  | *HapⅡ* | 0.683±0.009 |  |
|  | 2005LY | GL | *HapⅠ* | 0.627±0.007 | *** |
|  |  |  | *HapⅡ* | 0.696±0.007 |  |
|  | 2010SY | GL | *HapⅠ* | 0.6540.006 | *** |
|  |  |  | *HapⅡ* | 0.7.6±0.007 |  |
|  | 2002LY | GW | *HapⅠ* | 0.309±0.004 | *** |
|  |  |  | *HapⅡ* | 0.330±0.004 |  |
|  | 2005LY | GW | *HapⅠ* | 0.304±0.003 | *** |
|  |  |  | *HapⅡ* | 0.325±0.002 |  |
|  | 2010SY | GW | *HapⅠ* | 0.308±0.003 | *** |
|  |  |  | *HapⅡ* | 0.324±0.003 |  |
| *TaSINA111* | 2002LY | TGW | *HapⅠ* | 35.839±0.983 | *** |
|  |  |  | *HapⅡ* | 43.089±1.078 |  |
|  | 2005LY | TGW | *HapⅠ* | 33.031±0.947 | *** |
|  |  |  | *HapⅡ* | 39.829±1.109 |  |
|  | 2010SY | TGW | *HapⅠ* | 34.585±0.814 | *** |
|  |  |  | *HapⅡ* | 40.762±1.160 |  |
|  | 2002LY | GL | *HapⅠ* | 0.636±0.009 | ns |
|  |  |  | *HapⅡ* | 0.657±0.01 |  |
|  | 2005LY | GL | *HapⅠ* | 0.641±0.008 | ** |
|  |  |  | *HapⅡ* | 0.67±50.009 |  |
|  | 2010SY | GL | *HapⅠ* | 0.665±0.007 | ns |
|  |  |  | *HapⅡ* | 0.686±0.009 |  |
|  | 2002LY | GW | *HapⅠ* | 0.307±0.005 | ** |
|  |  |  | *HapⅡ* | 0.327±0.004 |  |
|  | 2005LY | GW | *HapⅠ* | 0.301±0.003 | *** |
|  |  |  | *HapⅡ* | 0.327±0.003 |  |
|  | 2010SY | GW | *HapⅠ* | 0.308±0.003 | ** |
|  |  |  | *HapⅡ* | 0.323±0.004 |  |

**Table S9. Nucleotide polymorphisms detected in *TaSINA101* gene**

| **No.** | **Accession** | **Allele** | **Haplotype** |
| --- | --- | --- | --- |
| 1 | 09-228-9-2-1-1-1-2 | T/T | *TaSINA101-HapⅠ* |
| 2 | 11-257-4--1 | T/T | *TaSINA101-HapⅠ* |
| 3 | 12-37-10-1-4 | T/T | *TaSINA101-HapⅠ* |
| 4 | 1R1 | T/T | *TaSINA101-HapⅠ* |
| 5 | 1R14 | T/T | *TaSINA101-HapⅠ* |
| 6 | 1R25 | T/T | *TaSINA101-HapⅠ* |
| 7 | 21-30 | T/T | *TaSINA101-HapⅠ* |
| 8 | 22-23 | T/T | *TaSINA101-HapⅠ* |
| 9 | 27-1 | T/T | *TaSINA101-HapⅠ* |
| 10 | 29-2-2 | T/T | *TaSINA101-HapⅠ* |
| 11 | 85-173-4 | T/T | *TaSINA101-HapⅠ* |
| 12 | 94164-1 | T/T | *TaSINA101-HapⅠ* |
| 13 | A8-4-4-2 | T/T | *TaSINA101-HapⅠ* |
| 14 | B11-2-3-1-1-2 | T/T | *TaSINA101-HapⅠ* |
| 15 | B160-2-2-1-1 | T/T | *TaSINA101-HapⅠ* |
| 16 | B17-2-3-2-1 | T/T | *TaSINA101-HapⅠ* |
| 17 | B61-2-3-2-1 | T/T | *TaSINA101-HapⅠ* |
| 18 | C14-14-1 | T/T | *TaSINA101-HapⅠ* |
| 19 | C47-1-1-2 | T/T | *TaSINA101-HapⅠ* |
| 20 | C53-3-2-1-1 | T/T | *TaSINA101-HapⅠ* |
| 21 | C72-6-1-2-1 | T/T | *TaSINA101-HapⅠ* |
| 22 | Dd52-17-3-2 | T/T | *TaSINA101-HapⅠ* |
| 23 | E69-4-1 | T/T | *TaSINA101-HapⅠ* |
| 24 | E71-2-6 | T/T | *TaSINA101-HapⅠ* |
| 25 | E72-2-2 | T/T | *TaSINA101-HapⅠ* |
| 26 | H-4-2-2-1 | T/T | *TaSINA101-HapⅠ* |
| 27 | Q9086 | T/T | *TaSINA101-HapⅠ* |
| 28 | Baiqimai | T/T | *TaSINA101-HapⅠ* |
| 29 | Bainong160 | T/T | *TaSINA101-HapⅠ* |
| 30 | Beijing837 | T/T | *TaSINA101-HapⅠ* |
| 31 | Beijing 8686 | T/T | *TaSINA101-HapⅠ* |
| 32 | Xifeng24 | T/T | *TaSINA101-HapⅠ* |
| 33 | Beinong2hao | T/T | *TaSINA101-HapⅠ* |
| 34 | Cangmai6001 | T/T | *TaSINA101-HapⅠ* |
| 35 | Changle5hao | T/T | *TaSINA101-HapⅠ* |
| 36 | Dongnong024 | T/T | *TaSINA101-HapⅠ* |
| 37 | Dong03-07 | T/T | *TaSINA101-HapⅠ* |
| 38 | Fengchan1 | T/T | *TaSINA101-HapⅠ* |
| 39 | Fuzhaung30 | T/T | *TaSINA101-HapⅠ* |
| 40 | Dan4589 | T/T | *TaSINA101-HapⅠ* |
| 41 | Dan6172 | T/T | *TaSINA101-HapⅠ* |
| 42 | Hanxuan1 | T/T | *TaSINA101-HapⅠ* |
| 43 | Hanxuan3 | T/T | *TaSINA101-HapⅠ* |
| 44 | Hangxuan01 | T/T | *TaSINA101-HapⅠ* |
| 45 | Hangxuan121 | T/T | *TaSINA101-HapⅠ* |
| 46 | Heng136 | T/T | *TaSINA101-HapⅠ* |
| 47 | Heng216 | T/T | *TaSINA101-HapⅠ* |
| 48 | Heng5229 | T/T | *TaSINA101-HapⅠ* |
| 49 | Hengguan35 | T/T | *TaSINA101-HapⅠ* |
| 50 | Hengshui6404 | T/T | *TaSINA101-HapⅠ* |
| 51 | Hengyou18 | T/T | *TaSINA101-HapⅠ* |
| 52 | Hongliang4 | T/T | *TaSINA101-HapⅠ* |
| 53 | Huabei187 | T/T | *TaSINA101-HapⅠ* |
| 54 | Huaimai25 | T/T | *TaSINA101-HapⅠ* |
| 55 | Huaishu10 | T/T | *TaSINA101-HapⅠ* |
| 56 | Jimai19 | T/T | *TaSINA101-HapⅠ* |
| 57 | Jimai229 | T/T | *TaSINA101-HapⅠ* |
| 58 | Jimai23 | T/T | *TaSINA101-HapⅠ* |
| 59 | Jimai44 | T/T | *TaSINA101-HapⅠ* |
| 60 | Jimai6hao | T/T | *TaSINA101-HapⅠ* |
| 61 | Jianan17 | T/T | *TaSINA101-HapⅠ* |
| 62 | Jimai29 | T/T | *TaSINA101-HapⅠ* |
| 63 | Jimai2 | T/T | *TaSINA101-HapⅠ* |
| 64 | Jimai32 | T/T | *TaSINA101-HapⅠ* |
| 65 | Jimai38 | T/T | *TaSINA101-HapⅠ* |
| 66 | Jimai6 | T/T | *TaSINA101-HapⅠ* |
| 67 | Jimai9 | T/T | *TaSINA101-HapⅠ* |
| 68 | Jian26 | T/T | *TaSINA101-HapⅠ* |
| 69 | Jin2148-7 | T/T | *TaSINA101-HapⅠ* |
| 70 | Pubing151 | T/T | *TaSINA101-HapⅠ* |
| 71 | Jinmai13 | T/T | *TaSINA101-HapⅠ* |
| 72 | Jinmai16 | T/T | *TaSINA101-HapⅠ* |
| 73 | Jinmai25 | T/T | *TaSINA101-HapⅠ* |
| 74 | Jinmai33 | T/T | *TaSINA101-HapⅠ* |
| 75 | Jinmai40 | T/T | *TaSINA101-HapⅠ* |
| 76 | Jinmai44 | T/T | *TaSINA101-HapⅠ* |
| 77 | Jinmai47 | T/T | *TaSINA101-HapⅠ* |
| 78 | Jinmai51 | T/T | *TaSINA101-HapⅠ* |
| 79 | Jinmai53 | T/T | *TaSINA101-HapⅠ* |
| 80 | Jinmai54 | T/T | *TaSINA101-HapⅠ* |
| 81 | Jinmai72 | T/T | *TaSINA101-HapⅠ* |
| 82 | Jinnong207 | T/T | *TaSINA101-HapⅠ* |
| 83 | Jing411 | T/T | *TaSINA101-HapⅠ* |
| 84 | Jingdong17 | T/T | *TaSINA101-HapⅠ* |
| 85 | Jingdong22 | T/T | *TaSINA101-HapⅠ* |
| 86 | Jingdong 83 Dong 65 | T/T | *TaSINA101-HapⅠ* |
| 87 | Jingdong8 | T/T | *TaSINA101-HapⅠ* |
| 88 | Kangzhuang974 | T/T | *TaSINA101-HapⅠ* |
| 89 | Kenong199 | T/T | *TaSINA101-HapⅠ* |
| 90 | Keyi26 | T/T | *TaSINA101-HapⅠ* |
| 91 | Keyi29 | T/T | *TaSINA101-HapⅠ* |
| 92 | Xifeng24 | T/T | *TaSINA101-HapⅠ* |
| 93 | Lantian10 | T/T | *TaSINA101-HapⅠ* |
| 94 | Lantian11 | T/T | *TaSINA101-HapⅠ* |
| 95 | Lantian13 | T/T | *TaSINA101-HapⅠ* |
| 96 | Lantian18 | T/T | *TaSINA101-HapⅠ* |
| 97 | Lantian21 | T/T | *TaSINA101-HapⅠ* |
| 98 | Lantian22 | T/T | *TaSINA101-HapⅠ* |
| 99 | Lantian28 | T/T | *TaSINA101-HapⅠ* |
| 100 | Lantian30 | T/T | *TaSINA101-HapⅠ* |
| 101 | Lantian33 | T/T | *TaSINA101-HapⅠ* |
| 102 | Lantian35 | T/T | *TaSINA101-HapⅠ* |
| 103 | Lantian4 | T/T | *TaSINA101-HapⅠ* |
| 104 | Lanxuan1 | T/T | *TaSINA101-HapⅠ* |
| 105 | Linfeng3 | T/T | *TaSINA101-HapⅠ* |
| 106 | Linhan234 | T/T | *TaSINA101-HapⅠ* |
| 107 | Longjian103 | T/T | *TaSINA101-HapⅠ* |
| 108 | Longjian107 | T/T | *TaSINA101-HapⅠ* |
| 109 | Longjian108 | T/T | *TaSINA101-HapⅠ* |
| 110 | Longjian110 | T/T | *TaSINA101-HapⅠ* |
| 111 | Longjian127 | T/T | *TaSINA101-HapⅠ* |
| 112 | Longjian386 | T/T | *TaSINA101-HapⅠ* |
| 113 | Longmai079 | T/T | *TaSINA101-HapⅠ* |
| 114 | Longmai838 | T/T | *TaSINA101-HapⅠ* |
| 115 | Longmai844 | T/T | *TaSINA101-HapⅠ* |
| 116 | Longyu218 | T/T | *TaSINA101-HapⅠ* |
| 117 | Longyuan235 | T/T | *TaSINA101-HapⅠ* |
| 118 | Longyuan937 | T/T | *TaSINA101-HapⅠ* |
| 119 | Longyuan961 | T/T | *TaSINA101-HapⅠ* |
| 120 | Longyuan036 | T/T | *TaSINA101-HapⅠ* |
| 121 | Longzhong1hao | T/T | *TaSINA101-HapⅠ* |
| 122 | Longzimai1 | T/T | *TaSINA101-HapⅠ* |
| 123 | Lumai14 | T/T | *TaSINA101-HapⅠ* |
| 124 | Xifeng24 | T/T | *TaSINA101-HapⅠ* |
| 125 | Lumai3 | T/T | *TaSINA101-HapⅠ* |
| 126 | Lunxuan987 | T/T | *TaSINA101-HapⅠ* |
| 127 | Luohan3 | T/T | *TaSINA101-HapⅠ* |
| 128 | Luohan8 | T/T | *TaSINA101-HapⅠ* |
| 129 | Luohan9hao | T/T | *TaSINA101-HapⅠ* |
| 130 | Luoyou7 | T/T | *TaSINA101-HapⅠ* |
| 131 | Xifeng24 | T/T | *TaSINA101-HapⅠ* |
| 132 | Ningmai12hao | T/T | *TaSINA101-HapⅠ* |
| 133 | Ningmai5 | T/T | *TaSINA101-HapⅠ* |
| 134 | Nongda135 | T/T | *TaSINA101-HapⅠ* |
| 135 | Pingliang35 | T/T | *TaSINA101-HapⅠ* |
| 136 | Qinmai3 | T/T | *TaSINA101-HapⅠ* |
| 137 | Qingmai7 | T/T | *TaSINA101-HapⅠ* |
| 138 | Qingnong4 | T/T | *TaSINA101-HapⅠ* |
| 139 | Qingshan851 | T/T | *TaSINA101-HapⅠ* |
| 140 | Qingshan782 | T/T | *TaSINA101-HapⅠ* |
| 141 | Qingshan843 | T/T | *TaSINA101-HapⅠ* |
| 142 | Shannongyoumai2 | T/T | *TaSINA101-HapⅠ* |
| 143 | Shanhan8675 | T/T | *TaSINA101-HapⅠ* |
| 144 | Shanyou225 | T/T | *TaSINA101-HapⅠ* |
| 145 | Shi4185 | T/T | *TaSINA101-HapⅠ* |
| 146 | Shimai12 | T/T | *TaSINA101-HapⅠ* |
| 147 | Shimai13 | T/T | *TaSINA101-HapⅠ* |
| 148 | Xifeng24 | T/T | *TaSINA101-HapⅠ* |
| 149 | Shimai18 | T/T | *TaSINA101-HapⅠ* |
| 150 | Shimai19 | T/T | *TaSINA101-HapⅠ* |
| 151 | Shiyou17 | T/T | *TaSINA101-HapⅠ* |
| 152 | Shiyou20 | T/T | *TaSINA101-HapⅠ* |
| 153 | Shunmai1718 | T/T | *TaSINA101-HapⅠ* |
| 154 | Silenghonghulutou | T/T | *TaSINA101-HapⅠ* |
| 155 | Tai13606 | T/T | *TaSINA101-HapⅠ* |
| 156 | Taiyuan633 | T/T | *TaSINA101-HapⅠ* |
| 157 | Wanmai 19 | T/T | *TaSINA101-HapⅠ* |
| 158 | Weimai4hao | T/T | *TaSINA101-HapⅠ* |
| 159 | Wenmai6hao | T/T | *TaSINA101-HapⅠ* |
| 160 | Xifeng16 | T/T | *TaSINA101-HapⅠ* |
| 161 | Xifeng20 | T/T | *TaSINA101-HapⅠ* |
| 162 | Xifeng24 | T/T | *TaSINA101-HapⅠ* |
| 163 | Xifeng27 | T/T | *TaSINA101-HapⅠ* |
| 164 | Xinong219 | T/T | *TaSINA101-HapⅠ* |
| 165 | Xinong688 | T/T | *TaSINA101-HapⅠ* |
| 166 | Xiannong4 | T/T | *TaSINA101-HapⅠ* |
| 167 | Xindong20 | T/T | *TaSINA101-HapⅠ* |
| 168 | Xindong22 | T/T | *TaSINA101-HapⅠ* |
| 169 | Xuzhou21 | T/T | *TaSINA101-HapⅠ* |
| 170 | Yannong19 | T/T | *TaSINA101-HapⅠ* |
| 171 | Yannong21 | T/T | *TaSINA101-HapⅠ* |
| 172 | Yumai13 | T/T | *TaSINA101-HapⅠ* |
| 173 | Yumai29 | T/T | *TaSINA101-HapⅠ* |
| 174 | Yumai2hao | T/T | *TaSINA101-HapⅠ* |
| 175 | Yunong949 | T/T | *TaSINA101-HapⅠ* |
| 176 | Yuandong3hao | T/T | *TaSINA101-HapⅠ* |
| 177 | Yunhan102 | T/T | *TaSINA101-HapⅠ* |
| 178 | Yunhan115 | T/T | *TaSINA101-HapⅠ* |
| 179 | Yunhan20410 | T/T | *TaSINA101-HapⅠ* |
| 180 | Yunhan21-30 | T/T | *TaSINA101-HapⅠ* |
| 181 | Yunhan22-33 | T/T | *TaSINA101-HapⅠ* |
| 182 | Yunhan618 | T/T | *TaSINA101-HapⅠ* |
| 183 | Yunhan719 | T/T | *TaSINA101-HapⅠ* |
| 184 | Chang6154 | T/T | *TaSINA101-HapⅠ* |
| 185 | Chang6359 | T/T | *TaSINA101-HapⅠ* |
| 186 | Chang6452 | T/T | *TaSINA101-HapⅠ* |
| 187 | Chang6878 | T/T | *TaSINA101-HapⅠ* |
| 188 | Chang7080 | T/T | *TaSINA101-HapⅠ* |
| 189 | Chang844 | T/T | *TaSINA101-HapⅠ* |
| 190 | Changzhi516 | T/T | *TaSINA101-HapⅠ* |
| 191 | Changzhi620 | T/T | *TaSINA101-HapⅠ* |
| 192 | Zhengfeng9962 | T/T | *TaSINA101-HapⅠ* |
| 193 | Zhenzhou24 | T/T | *TaSINA101-HapⅠ* |
| 194 | Zhong7902 | T/T | *TaSINA101-HapⅠ* |
| 195 | Zhongmai175 | T/T | *TaSINA101-HapⅠ* |
| 196 | Zhongmai36 | T/T | *TaSINA101-HapⅠ* |
| 197 | Zhongmai9hao | T/T | *TaSINA101-HapⅠ* |
| 198 | Zhongyou9507 | T/T | *TaSINA101-HapⅠ* |
| 199 | Zhoumai18 | T/T | *TaSINA101-HapⅠ* |
| 200 | Zhoumai22 | T/T | *TaSINA101-HapⅠ* |
| 201 | Zhoumai23 | T/T | *TaSINA101-HapⅠ* |
| 202 | 0025-17-1 | G/G | *TaSINA101-HapⅠI* |
| 203 | 00-71 | G/G | *TaSINA101-HapⅠI* |
| 204 | 01-368 | G/G | *TaSINA101-HapⅠI* |
| 205 | 02-7 | G/G | *TaSINA101-HapⅠI* |
| 206 | 10-160-6-1-1-5-2-3 | G/G | *TaSINA101-HapⅠI* |
| 207 | 1389-2 | G/G | *TaSINA101-HapⅠI* |
| 208 | 9840-0-3-2 | G/G | *TaSINA101-HapⅠI* |
| 209 | 988-4-2-4-1 | G/G | *TaSINA101-HapⅠI* |
| 210 | 99384-2-1 | G/G | *TaSINA101-HapⅠI* |
| 211 | A80-3-1-1-3 | G/G | *TaSINA101-HapⅠI* |
| 212 | C130-5-1-1 | G/G | *TaSINA101-HapⅠI* |
| 213 | C42-2-3-3 | G/G | *TaSINA101-HapⅠI* |
| 214 | C49-1-1-1-2 | G/G | *TaSINA101-HapⅠI* |
| 215 | C78-3-6-1-3 | G/G | *TaSINA101-HapⅠI* |
| 216 | Fanmai8 | G/G | *TaSINA101-HapⅠI* |
| 217 | Huaimai18 | G/G | *TaSINA101-HapⅠI* |
| 218 | Jimai20 | G/G | *TaSINA101-HapⅠI* |
| 219 | Jimai262 | G/G | *TaSINA101-HapⅠI* |
| 220 | Jimai4 | G/G | *TaSINA101-HapⅠI* |
| 221 | Jinmai17 | G/G | *TaSINA101-HapⅠI* |
| 222 | Xifeng24 | G/G | *TaSINA101-HapⅠI* |
| 223 | Jingdong 82 Dong 307 | G/G | *TaSINA101-HapⅠI* |
| 224 | Jinghua1 | G/G | *TaSINA101-HapⅠI* |
| 225 | Jingshuang2hao | G/G | *TaSINA101-HapⅠI* |
| 226 | Lantain14 | G/G | *TaSINA101-HapⅠI* |
| 227 | Lantian15 | G/G | *TaSINA101-HapⅠI* |
| 228 | Lin138 | G/G | *TaSINA101-HapⅠI* |
| 229 | Linhan538 | G/G | *TaSINA101-HapⅠI* |
| 230 | Longjian111 | G/G | *TaSINA101-HapⅠI* |
| 231 | Longjian294 | G/G | *TaSINA101-HapⅠI* |
| 232 | Longmai847 | G/G | *TaSINA101-HapⅠI* |
| 233 | Longyu5hao | G/G | *TaSINA101-HapⅠI* |
| 234 | Longzhong2hao | G/G | *TaSINA101-HapⅠI* |
| 235 | Lumai17 | G/G | *TaSINA101-HapⅠI* |
| 236 | Lumai5 | G/G | *TaSINA101-HapⅠI* |
| 237 | Luohan11 | G/G | *TaSINA101-HapⅠI* |
| 238 | Luohan2 | G/G | *TaSINA101-HapⅠI* |
| 239 | Luohan6 | G/G | *TaSINA101-HapⅠI* |
| 240 | Luohan7 | G/G | *TaSINA101-HapⅠI* |
| 241 | Luomai22 | G/G | *TaSINA101-HapⅠI* |
| 242 | Luomai23 | G/G | *TaSINA101-HapⅠI* |
| 243 | Luomai8 | G/G | *TaSINA101-HapⅠI* |
| 244 | Qingnong3 | G/G | *TaSINA101-HapⅠI* |
| 245 | Shannongfu63 | G/G | *TaSINA101-HapⅠI* |
| 246 | Taishan1hao | G/G | *TaSINA101-HapⅠI* |
| 247 | Xinong1043 | G/G | *TaSINA101-HapⅠI* |
| 248 | Xinmai296 | G/G | *TaSINA101-HapⅠI* |
| 249 | Yunong 416 | G/G | *TaSINA101-HapⅠI* |
| 250 | Yuzhan4 | G/G | *TaSINA101-HapⅠI* |
| 251 | Yunhan2028 | G/G | *TaSINA101-HapⅠI* |
| 252 | Yunhan805 | G/G | *TaSINA101-HapⅠI* |
| 253 | Chang8744 | G/G | *TaSINA101-HapⅠI* |
| 254 | Chang4738 | G/G | *TaSINA101-HapⅠI* |
| 255 | Changqu521 | G/G | *TaSINA101-HapⅠI* |
| 256 | Zhongmai553 | G/G | *TaSINA101-HapⅠI* |

**Table S10. Nucleotide polymorphisms detected in *TaSINA109* gene**

| **No.** | **Accession** | **Allele** | **Haplotype** |
| --- | --- | --- | --- |
| 1 | 0025-17-1 | T | *TaSINA109-HapI* |
| 2 | 01-368 | T | *TaSINA109-HapI* |
| 3 | 02-7 | T | *TaSINA109-HapI* |
| 4 | 09-228-9-2-1-1-1-2 | T | *TaSINA109-HapI* |
| 5 | 12-37-10-1-4 | T | *TaSINA109-HapI* |
| 6 | 85-173-4 | T | *TaSINA109-HapI* |
| 7 | 99384-2-1 | T | *TaSINA109-HapI* |
| 8 | A8-4-4-2 | T | *TaSINA109-HapI* |
| 9 | C130-5-1-1 | T | *TaSINA109-HapI* |
| 10 | E69-4-1 | T | *TaSINA109-HapI* |
| 11 | Changle5hao | T | *TaSINA109-HapI* |
| 12 | Dongxie2 | T | *TaSINA109-HapI* |
| 13 | Fengchan1 | T | *TaSINA109-HapI* |
| 14 | Fuzhaung30 | T | *TaSINA109-HapI* |
| 15 | Dan6172 | T | *TaSINA109-HapI* |
| 16 | Hanxuan10hao | T | *TaSINA109-HapI* |
| 17 | Hanxuan2hao | T | *TaSINA109-HapI* |
| 18 | Heng216 | T | *TaSINA109-HapI* |
| 19 | Heng4399 | T | *TaSINA109-HapI* |
| 20 | Hengguan35 | T | *TaSINA109-HapI* |
| 21 | Huapei6 | T | *TaSINA109-HapI* |
| 22 | Huaimai25 | T | *TaSINA109-HapI* |
| 23 | Jimai20 | T | *TaSINA109-HapI* |
| 24 | Jimai21 | T | *TaSINA109-HapI* |
| 25 | Jimai4 | T | *TaSINA109-HapI* |
| 26 | Jimai29 | T | *TaSINA109-HapI* |
| 27 | Jimai2 | T | *TaSINA109-HapI* |
| 28 | Jimai30 | T | *TaSINA109-HapI* |
| 29 | Jimai6 | T | *TaSINA109-HapI* |
| 30 | Jimai9 | T | *TaSINA109-HapI* |
| 31 | Jinmai16 | T | *TaSINA109-HapI* |
| 32 | Jinmai17 | T | *TaSINA109-HapI* |
| 33 | Jinmai39 | T | *TaSINA109-HapI* |
| 34 | Jinmai44 | T | *TaSINA109-HapI* |
| 35 | Jinmai51 | T | *TaSINA109-HapI* |
| 36 | Jinmai63 | T | *TaSINA109-HapI* |
| 37 | Xifeng24 | T | *TaSINA109-HapI* |
| 38 | Jingdong17 | T | *TaSINA109-HapI* |
| 39 | Jingdong22 | T | *TaSINA109-HapI* |
| 40 | Jingdong 82 Dong 307 | T | *TaSINA109-HapI* |
| 41 | Jinghua1 | T | *TaSINA109-HapI* |
| 42 | Kangzhuang974 | T | *TaSINA109-HapI* |
| 43 | Lantian11 | T | *TaSINA109-HapI* |
| 44 | Lantain14 | T | *TaSINA109-HapI* |
| 45 | Lantian15 | T | *TaSINA109-HapI* |
| 46 | Lantian18 | T | *TaSINA109-HapI* |
| 47 | Lantian35 | T | *TaSINA109-HapI* |
| 48 | Lin138 | T | *TaSINA109-HapI* |
| 49 | Linhan538 | T | *TaSINA109-HapI* |
| 50 | Longmai847 | T | *TaSINA109-HapI* |
| 51 | Longyu5hao | T | *TaSINA109-HapI* |
| 52 | Longyuan235 | T | *TaSINA109-HapI* |
| 53 | Longyuan961 | T | *TaSINA109-HapI* |
| 54 | Longzhong1hao | T | *TaSINA109-HapI* |
| 55 | Longzhong2hao | T | *TaSINA109-HapI* |
| 56 | Lumai5 | T | *TaSINA109-HapI* |
| 57 | Luohan8 | T | *TaSINA109-HapI* |
| 58 | Luomai9 | T | *TaSINA109-HapI* |
| 59 | Qinmai3 | T | *TaSINA109-HapI* |
| 60 | Qingmai7 | T | *TaSINA109-HapI* |
| 61 | Qingnong3 | T | *TaSINA109-HapI* |
| 62 | Qingnong4 | T | *TaSINA109-HapI* |
| 63 | Shimai18 | T | *TaSINA109-HapI* |
| 64 | Shimai19 | T | *TaSINA109-HapI* |
| 65 | Shiyou17 | T | *TaSINA109-HapI* |
| 66 | Taiyuan633 | T | *TaSINA109-HapI* |
| 67 | Wanmai 19 | T | *TaSINA109-HapI* |
| 68 | Xinong1043 | T | *TaSINA109-HapI* |
| 69 | Xinong219 | T | *TaSINA109-HapI* |
| 70 | Xindong18 | T | *TaSINA109-HapI* |
| 71 | Xindong20 | T | *TaSINA109-HapI* |
| 72 | Xindong22 | T | *TaSINA109-HapI* |
| 73 | Yannong21 | T | *TaSINA109-HapI* |
| 74 | Yanan15 | T | *TaSINA109-HapI* |
| 75 | Yumai29 | T | *TaSINA109-HapI* |
| 76 | Yumai2hao | T | *TaSINA109-HapI* |
| 77 | Yunong 416 | T | *TaSINA109-HapI* |
| 78 | Yunong949 | T | *TaSINA109-HapI* |
| 79 | Yuzhan4 | T | *TaSINA109-HapI* |
| 80 | Yuandong3hao | T | *TaSINA109-HapI* |
| 81 | Yunhan719 | T | *TaSINA109-HapI* |
| 82 | Chang4738 | T | *TaSINA109-HapI* |
| 83 | Chang6359 | T | *TaSINA109-HapI* |
| 84 | Chang7080 | T | *TaSINA109-HapI* |
| 85 | Changzhi620 | T | *TaSINA109-HapI* |
| 86 | Zhoumai23 | T | *TaSINA109-HapI* |
| 87 | 10-160-6-1-1-5-2-3 | C | *TaSINA109-HapII* |
| 88 | 11-257-4--1 | C | *TaSINA109-HapII* |
| 89 | 1R1 | C | *TaSINA109-HapII* |
| 90 | 1R25 | C | *TaSINA109-HapII* |
| 91 | 21-30 | C | *TaSINA109-HapII* |
| 92 | 22-23 | C | *TaSINA109-HapII* |
| 93 | 27-1 | C | *TaSINA109-HapII* |
| 94 | 29-2-2 | C | *TaSINA109-HapII* |
| 95 | 94164-1 | C | *TaSINA109-HapII* |
| 96 | 980-4-1-1-2 | C | *TaSINA109-HapII* |
| 97 | 988-4-2-4-1 | C | *TaSINA109-HapII* |
| 98 | A80-3-1-1-3 | C | *TaSINA109-HapII* |
| 99 | B11-2-3-1-1-2 | C | *TaSINA109-HapII* |
| 100 | B160-2-2-1-1 | C | *TaSINA109-HapII* |
| 101 | B61-2-3-2-1 | C | *TaSINA109-HapII* |
| 102 | C14-14-1 | C | *TaSINA109-HapII* |
| 103 | C42-2-3-3 | C | *TaSINA109-HapII* |
| 104 | C47-1-1-2 | C | *TaSINA109-HapII* |
| 105 | C49-1-1-1-2 | C | *TaSINA109-HapII* |
| 106 | C53-3-2-1-1 | C | *TaSINA109-HapII* |
| 107 | C78-3-6-1-3 | C | *TaSINA109-HapII* |
| 108 | Dd52-17-3-2 | C | *TaSINA109-HapII* |
| 109 | E71-2-6 | C | *TaSINA109-HapII* |
| 110 | E72-2-2 | C | *TaSINA109-HapII* |
| 111 | H-4-2-2-1 | C | *TaSINA109-HapII* |
| 112 | Q9086 | C | *TaSINA109-HapII* |
| 113 | Bainong160 | C | *TaSINA109-HapII* |
| 114 | Beijing837 | C | *TaSINA109-HapII* |
| 115 | Beijing 8686 | C | *TaSINA109-HapII* |
| 116 | Xifeng24 | C | *TaSINA109-HapII* |
| 117 | Beinong2hao | C | *TaSINA109-HapII* |
| 118 | Boai7023 | C | *TaSINA109-HapII* |
| 119 | Dong03-07 | C | *TaSINA109-HapII* |
| 120 | Fanmai8 | C | *TaSINA109-HapII* |
| 121 | Hanxuan1 | C | *TaSINA109-HapII* |
| 122 | Hangxuan01 | C | *TaSINA109-HapII* |
| 123 | Hangxuan121 | C | *TaSINA109-HapII* |
| 124 | Hengshui6404 | C | *TaSINA109-HapII* |
| 125 | Hongliang4 | C | *TaSINA109-HapII* |
| 126 | Huabei187 | C | *TaSINA109-HapII* |
| 127 | Huaimai18 | C | *TaSINA109-HapII* |
| 128 | Jimai19 | C | *TaSINA109-HapII* |
| 129 | Jimai23 | C | *TaSINA109-HapII* |
| 130 | Jimai44 | C | *TaSINA109-HapII* |
| 131 | Jimai6hao | C | *TaSINA109-HapII* |
| 132 | Jinan13 | C | *TaSINA109-HapII* |
| 133 | Jianan17 | C | *TaSINA109-HapII* |
| 134 | Jimai26 | C | *TaSINA109-HapII* |
| 135 | Jimai38 | C | *TaSINA109-HapII* |
| 136 | Jin2148-7 | C | *TaSINA109-HapII* |
| 137 | Pubing151 | C | *TaSINA109-HapII* |
| 138 | Jinmai13 | C | *TaSINA109-HapII* |
| 139 | Jinmai25 | C | *TaSINA109-HapII* |
| 140 | Jinmai33 | C | *TaSINA109-HapII* |
| 141 | Jinmai40 | C | *TaSINA109-HapII* |
| 142 | Jinmai47 | C | *TaSINA109-HapII* |
| 143 | Jinmai50 | C | *TaSINA109-HapII* |
| 144 | Jinmai54 | C | *TaSINA109-HapII* |
| 145 | Jinmai72 | C | *TaSINA109-HapII* |
| 146 | Jing411 | C | *TaSINA109-HapII* |
| 147 | Jingdong8 | C | *TaSINA109-HapII* |
| 148 | Jingshuang16 | C | *TaSINA109-HapII* |
| 149 | Jingshuang2hao | C | *TaSINA109-HapII* |
| 150 | Xifeng24 | C | *TaSINA109-HapII* |
| 151 | Lantian10 | C | *TaSINA109-HapII* |
| 152 | Lantian13 | C | *TaSINA109-HapII* |
| 153 | Lantian21 | C | *TaSINA109-HapII* |
| 154 | Lantian22 | C | *TaSINA109-HapII* |
| 155 | Lantian2 | C | *TaSINA109-HapII* |
| 156 | Liangxing99 | C | *TaSINA109-HapII* |
| 157 | Linfeng3 | C | *TaSINA109-HapII* |
| 158 | Linfeng518 | C | *TaSINA109-HapII* |
| 159 | Linfeng615 | C | *TaSINA109-HapII* |
| 160 | Linhan51241 | C | *TaSINA109-HapII* |
| 161 | Linhan6hao | C | *TaSINA109-HapII* |
| 162 | Longjian101 | C | *TaSINA109-HapII* |
| 163 | Longjian103 | C | *TaSINA109-HapII* |
| 164 | Longjian107 | C | *TaSINA109-HapII* |
| 165 | Longjian108 | C | *TaSINA109-HapII* |
| 166 | Longjian110 | C | *TaSINA109-HapII* |
| 167 | Longjian111 | C | *TaSINA109-HapII* |
| 168 | Longmai079 | C | *TaSINA109-HapII* |
| 169 | Longmai838 | C | *TaSINA109-HapII* |
| 170 | Longmai844 | C | *TaSINA109-HapII* |
| 171 | Longyu218 | C | *TaSINA109-HapII* |
| 172 | Longyuan937 | C | *TaSINA109-HapII* |
| 173 | Longyuan036 | C | *TaSINA109-HapII* |
| 174 | Longzimai1 | C | *TaSINA109-HapII* |
| 175 | Lude1hao | C | *TaSINA109-HapII* |
| 176 | Lumai14 | C | *TaSINA109-HapII* |
| 177 | Xifeng24 | C | *TaSINA109-HapII* |
| 178 | Lumai1hao | C | *TaSINA109-HapII* |
| 179 | Lumai3 | C | *TaSINA109-HapII* |
| 180 | Lunxuan987 | C | *TaSINA109-HapII* |
| 181 | Luohan2 | C | *TaSINA109-HapII* |
| 182 | Luohan9hao | C | *TaSINA109-HapII* |
| 183 | Luomai22 | C | *TaSINA109-HapII* |
| 184 | Luomai23 | C | *TaSINA109-HapII* |
| 185 | Luomai8 | C | *TaSINA109-HapII* |
| 186 | Luoyou7 | C | *TaSINA109-HapII* |
| 187 | Neimai11 | C | *TaSINA109-HapII* |
| 188 | Xifeng24 | C | *TaSINA109-HapII* |
| 189 | Ningmai12hao | C | *TaSINA109-HapII* |
| 190 | Nongda135 | C | *TaSINA109-HapII* |
| 191 | Xifeng24 | C | *TaSINA109-HapII* |
| 192 | Nongda311 | C | *TaSINA109-HapII* |
| 193 | Pingliang35 | C | *TaSINA109-HapII* |
| 194 | Qingshan851 | C | *TaSINA109-HapII* |
| 195 | Qingshan782 | C | *TaSINA109-HapII* |
| 196 | Qingshan821 | C | *TaSINA109-HapII* |
| 197 | Qingshan843 | C | *TaSINA109-HapII* |
| 198 | Shannongfu63 | C | *TaSINA109-HapII* |
| 199 | Shannongyoumai2 | C | *TaSINA109-HapII* |
| 200 | Shanhan8675 | C | *TaSINA109-HapII* |
| 201 | Shijiazhuang8hao | C | *TaSINA109-HapII* |
| 202 | Shimai12 | C | *TaSINA109-HapII* |
| 203 | Shimai13 | C | *TaSINA109-HapII* |
| 204 | Shiyou20 | C | *TaSINA109-HapII* |
| 205 | Shunmai1718 | C | *TaSINA109-HapII* |
| 206 | Tai13606 | C | *TaSINA109-HapII* |
| 207 | Taishan1hao | C | *TaSINA109-HapII* |
| 208 | Taishan23 | C | *TaSINA109-HapII* |
| 209 | Tao157 | C | *TaSINA109-HapII* |
| 210 | Wenmai6hao | C | *TaSINA109-HapII* |
| 211 | Xifeng16 | C | *TaSINA109-HapII* |
| 212 | Xifeng20 | C | *TaSINA109-HapII* |
| 213 | Xifeng24 | C | *TaSINA109-HapII* |
| 214 | Xifeng27 | C | *TaSINA109-HapII* |
| 215 | Xifrng28 | C | *TaSINA109-HapII* |
| 216 | Xinong1018 | C | *TaSINA109-HapII* |
| 217 | Xinong189 | C | *TaSINA109-HapII* |
| 218 | Xinong318 | C | *TaSINA109-HapII* |
| 219 | Xinong688 | C | *TaSINA109-HapII* |
| 220 | Xinong979 | C | *TaSINA109-HapII* |
| 221 | Xinong979 | C | *TaSINA109-HapII* |
| 222 | Xiannong4 | C | *TaSINA109-HapII* |
| 223 | Xinmai296 | C | *TaSINA109-HapII* |
| 224 | Xuzhou21 | C | *TaSINA109-HapII* |
| 225 | Yannong19 | C | *TaSINA109-HapII* |
| 226 | Yanzhan1hao | C | *TaSINA109-HapII* |
| 227 | Yumai13 | C | *TaSINA109-HapII* |
| 228 | Yumai18 | C | *TaSINA109-HapII* |
| 229 | Yunhan102 | C | *TaSINA109-HapII* |
| 230 | Yunhan2028 | C | *TaSINA109-HapII* |
| 231 | Yunhan20410 | C | *TaSINA109-HapII* |
| 232 | Yunhan21-30 | C | *TaSINA109-HapII* |
| 233 | Yunhan22-33 | C | *TaSINA109-HapII* |
| 234 | Yunhan618 | C | *TaSINA109-HapII* |
| 235 | Yunhan805 | C | *TaSINA109-HapII* |
| 236 | Chang4640 | C | *TaSINA109-HapII* |
| 237 | Chang6154 | C | *TaSINA109-HapII* |
| 238 | Chang6452 | C | *TaSINA109-HapII* |
| 239 | Chang6878 | C | *TaSINA109-HapII* |
| 240 | Chang844 | C | *TaSINA109-HapII* |
| 241 | Chang4738 | C | *TaSINA109-HapII* |
| 242 | Changzhi516 | C | *TaSINA109-HapII* |
| 243 | Changzhi6406 | C | *TaSINA109-HapII* |
| 244 | Zhengfeng9962 | C | *TaSINA109-HapII* |
| 245 | Zhenzhou24 | C | *TaSINA109-HapII* |
| 246 | Zhong7902 | C | *TaSINA109-HapII* |
| 247 | Zhongmai175 | C | *TaSINA109-HapII* |
| 248 | Zhongmai36 | C | *TaSINA109-HapII* |
| 249 | Zhongmai9hao | C | *TaSINA109-HapII* |
| 250 | Zhongyou9507 | C | *TaSINA109-HapII* |
| 251 | Zhoumai16 | C | *TaSINA109-HapII* |
| 252 | Zhoumai18 | C | *TaSINA109-HapII* |
| 253 | Zhoumai22 | C | *TaSINA109-HapII* |
