# Supplementary material for: Genomic‐wide analysis reveals seven in absentia genes regulating grain development in wheat (Triticum aestivum L.)
Source: Plant Genome. 2024 Jun 5;17(3):e20480. doi: 10.1002/tpg2.20480 (PMC12807368; doi:10.1002/tpg2.20480)
Supplement: Supplementary file 2 [file TPG2-17-e20480-s002.docx]

**Figure S1** The frequency of different haplotypes in TaSINA genes of different TaSINA family subclades. The number of haplotypes of the TaSINA family genes is represented in different colors.

**Figure S2.** The morphology of *TaSINA101* overexpression lines and wild type ZH11 in the mature stage. Bar =10 cm.
